# Subtype-specific kinase dependency regulates growth and metastasis of poor-prognosis mesenchymal colorectal cancer

**DOI:** 10.1186/s13046-023-02600-9

**Published:** 2023-03-03

**Authors:** Joyce Y. Buikhuisen, Patricia M. Gomez Barila, Kate Cameron, Saskia J. E. Suijkerbuijk, Cor Lieftink, Simone di Franco, Ana Krotenberg Garcia, Rebeca Uceda Castro, Kristiaan J. Lenos, Lisanne E. Nijman, Arezo Torang, Ciro Longobardi, Joan H. de Jong, Daniëlle Dekker, Giorgio Stassi, Louis Vermeulen, Roderick L. Beijersbergen, Jacco van Rheenen, Stephan Huveneers, Jan Paul Medema

**Affiliations:** 1 Laboratory for Experimental Oncology and Radiobiology, Center for Experimental Molecular Medicine, Cancer Center Amsterdam, Amsterdam UMC, University of Amsterdam, location AMC, Meibergdreef 9, 1105 AZ Amsterdam, The Netherlands; 2 Oncode Institute, Amsterdam, The Netherlands; 3 Department of Molecular Pathology, The Netherlands Cancer Institute, Amsterdam, The Netherlands; 4 Division of Molecular Carcinogenesis, The Netherlands Cancer Institute, Amsterdam, The Netherlands; 5 Department of Surgical Oncological and Stomatological Sciences (DICHIRONS), University of Palermo, Palermo, Italy; 6 Department of Medical Biochemistry, Amsterdam Cardiovascular Sciences, Amsterdam UMC, University of Amsterdam, Amsterdam, The Netherlands

**Keywords:** Colorectal cancer, Epithelial-mesenchymal transition, Metastasis, Cellular attachment, PAK family

## Abstract

**Background:**

Colorectal cancer (CRC) can be divided into four consensus molecular subtypes (CMS), each with distinct biological features. CMS4 is associated with epithelial-mesenchymal transition and stromal infiltration (Guinney et al., Nat Med 21:1350–6, 2015; Linnekamp et al., Cell Death Differ 25:616–33, 2018), whereas clinically it is characterized by lower responses to adjuvant therapy, higher incidence of metastatic spreading and hence dismal prognosis (Buikhuisen et al., Oncogenesis 9:66, 2020).

**Methods:**

To understand the biology of the mesenchymal subtype and unveil specific vulnerabilities, a large CRISPR-Cas9 drop-out screen was performed on 14 subtyped CRC cell lines to uncover essential kinases in all CMSs. Dependency of CMS4 cells on p21-activated kinase 2 (PAK2) was validated in independent 2D and 3D in vitro cultures and in vivo models assessing primary and metastatic outgrowth in liver and peritoneum. TIRF microscopy was used to uncover actin cytoskeleton dynamics and focal adhesion localization upon PAK2 loss. Subsequent functional assays were performed to determine altered growth and invasion patterns.

**Results:**

PAK2 was identified as a key kinase uniquely required for growth of the mesenchymal subtype CMS4, both in vitro and in vivo. PAK2 plays an important role in cellular attachment and cytoskeletal rearrangements (Coniglio et al., Mol Cell Biol 28:4162–72, 2008; Grebenova et al., Sci Rep 9:17171, 2019). In agreement, deletion or inhibition of PAK2 impaired actin cytoskeleton dynamics in CMS4 cells and, as a consequence, significantly reduced invasive capacity, while it was dispensable for CMS2 cells. Clinical relevance of these findings was supported by the observation that deletion of PAK2 from CMS4 cells prevented metastatic spreading in vivo. Moreover, growth in a model for peritoneal metastasis was hampered when CMS4 tumor cells were deficient for PAK2.

**Conclusion:**

Our data reveal a unique dependency of mesenchymal CRC and provide a rationale for PAK2 inhibition to target this aggressive subgroup of colorectal cancer.

**Supplementary Information:**

The online version contains supplementary material available at 10.1186/s13046-023-02600-9.

## Background

Colorectal cancer (CRC) is a heterogeneous disease at both the molecular and clinical level. There are two major groups by which CRC is classically identified: the chromosomal instable (CIN) and microsatellite instable (MSI), the former encompassing the majority of cases [1, 2]. Although this classification system is clinically insightful to some extent, it does not fully explain the differences in treatment responses or prognosis, and neither do common mutations or dysregulated pathways [3]. Therefore, mRNA expression-based classification of CRC was employed to capture the heterogeneity present in this disease and this has resulted in the definition of consensus molecular subtypes (CMSs) [1, 2, 4]. CMS classification recognizes MSI tumors mainly as CMS1, whilst the majority of CIN tumors are subdivided over CMS2-4. Part of the distinct gene expression patterns found in each subtype can be attributed to infiltration or presence of a uniquely shaped microenvironment. Specifically, CMS4 cancers are characterized by an abundant stromal infiltrate, mainly consisting of fibroblasts, which contributes to the mesenchymal gene expression program in this subtype, while CMS1 is defined by high immune infiltrate [5, 6]. Importantly, not only do the four subtypes possess clearly distinct biological features, they also follow a different clinical course. Specifically, the mesenchymal subgroup CMS4 has received attention due to its enhanced capacity of metastatic spreading and its poor response to therapy [7]. We and others have shown that in vitro cultures retain a large part of the differential CMS pathway activity found in tumors even though the gene expression programs emanating from the stromal compartment are lacking in this setting. Tumor cell intrinsic gene expression differences are thus translated to (primary) cell cultures, allowing for the identification of all 4 subtypes in CRC pre-clinical models [8–10]. Supporting this notion is the fact that growth patterns of mesenchymal CMS4 CRC cell lines are evidently distinct from the more epithelial CMS2 and CMS3 lines and on average show less cell-to-cell contact and a more elongated phenotype [8, 10]. In addition, CMS4 is associated strongly with metastatic spreading in in vivo mouse models [11–13].

We hypothesized that these biologically distinct features of CRC subtypes translate into unique dependencies for growth and survival. To identify CMS-selective sensitivities, a CRISPR-Cas9-based drop-out screen targeting the kinome was performed. Using this approach, multiple CMS-specific essential kinases were identified, confirming the distinct wiring of the subtypes. Subsequently, we zoomed in on dependencies identified in the aggressive CMS4 cell lines and validated the top hit, PAK2, to be a CMS4-specific drop-out crucial for outgrowth of these lines both in vitro and in vivo*.* Importantly, PAK2 was not differentially expressed between subtypes and the dependency of the mesenchymal lines on this kinase was shown to relate to its essential role in actin cytoskeletal remodeling. Additionally, CMS4 lines were more sensitive to chemical PAK inhibition and require PAK2 for invasion and in vivo metastasis.

## Materials and methods

### Cell and organoid culture, lentivirus packaging and transduction

Human colon cancer cell lines were a kind gift from the Sanger Institute (Cambridge, United Kingdom). CaR-1, HT55, Hutu-80, LS123, LS180, OUMS-23, RKO, SW48 and SW948 were cultured in 1:1 DMEM/F12 medium containing 15 mM HEPES and 2,5 mM L-glutamine (Cat. #31330095, Gibco) supplemented with 8% fetal bovine serum (FBS) (Cat. #s-FBS-SA-025, Serana) and 50 units/mL of penicillin/streptomycin (Cat. #15140122, Gibco). Colo320-HSR, HCT116, KM12, LS513, LS1034, MDST8 and RCM-1 were maintained in RPMI 1640 medium containing 25 mM HEPES and 2,05 mM L-glutamine (Cat. #52400041, Gibco) supplemented with 8% FBS, 1% D-glucose solution plus (Cat. # G8769, Sigma-Aldrich), 1 mM sodium pyruvate (Cat. #11360070, Gibco) and 50 units/mL of penicillin/streptomycin. HEK293T (ATCC) were cultured in DMEM medium (Cat. # BE12-614F, Lonza), supplemented with 8% FBS, glutamine and 50 units/mL of penicillin/streptomycin. All lines were cultured in a humidified incubator at 37 °C with 5% CO_2_. Phase contrast images of CRC cell lines were obtained on a Nikon Eclipse TS100 (HuTu-80, MDST8, OUMS23; Nikon Microscopy) and an EVOS FL auto (HT55; Life Technologies).

Human organoids p11t and p26t were cultured according to the protocol previously described by van de Wetering, et al. [14]. Organoids were dissociated once a week using TrypLE (Cat. # 12604013, ThermoFisher Scientific) and maintained in culture in a humidified incubator at 37 °C with 5% CO_2_. Primary spheroid cultures CONC and Co108 were obtained and cultured in low-adherent plates in cancer stem cell medium as previously described [8]. Briefly, advanced DMEM/F12 medium was supplemented with D-glucose 0.15% (Sigma), HEPES 5 mM (Life Technologies), penicillin/streptomycin (PS), Heparin 2 μg/ml (Sigma), Insulin 10 μg/ml (Sigma), β-mercaptoethanol 100 μM (Sigma), and trace element B and C (Fisher Scientific) with freshly added human EGF 20 ng/ml (Peprotech) and FGF 10 ng/ml. For the pTRIPZ transduced cell lines, Doxycycline was added to the medium at a concentration of 1 μg/ml for at least 3 days before performing experiments.

Cell lines were authenticated by short-tandem-repeat DNA profiling using the using the PowerPlex 16® system (Cat. #DC6531, Promega) according to the manufacturer’s protocol. Results were analyzed using GeneScan® software (Applied Biosystems). Mycoplasma tests were performed once a month, positive cultures were immediately excluded from analysis and further use in experiments.

Plasmids used for this work are listed below, including the original source from which they were obtained.

**Table:** 

Plasmid name	Source	Catalogue number/reference
psPAX2	Addgene	12,260
pMD2.G	Addgene	12,259
LentiCRISPRv2.1 kinome library	Provided by Beijersbergen lab	52,961 (backbone)
pTRIPZ shRNA non-silencing control	Horizon Discovery	RHS4743
pTRIPZ shRNA PAK2 (sh-1)	Horizon Discovery	Clone V3THS_315625, RHS4696-200771936
pSpCas9(BB)-2A-GFP (pX458)	Addgene	48,138
pLKO.1 non-silencing control	MISSION® TRC library, Merck	SHC002
pLKO.1 ARHGEF7 shRNA-1	Provided by Versteeg lab	TRCN0000047593
pLKO.1 ARHGEF7 shRNA-2	Provided by Versteeg lab	TRCN0000047596
pRRL-Lifeact-GFP	Provided by Huveneers lab	
pRRL-paxillin-mCherry	Provided by Huveneers lab	
pLV.CMV.H2B-Dendra2-bc-puro	Provided by van Rheenen lab	Alieva M. et al. 2017 Sci. Rep [15].
LeGO-T2 (tdTomato)	Addgene	27,342

Lentivirus was produced in HEK293T cells using second generation packaging plasmids psPAX2 and pMD2.G. Cells were transfected overnight with 1 mg/ml PEI in a 1:2 plasmid:PEI ratio. Viral supernatant was collected 2 and 3 days after transfection, pooled and filtered (0,45 μm). All transductions, except for transduction with the lentiCRISPRv2.1 kinome library (specified below) were carried out as follows: CRC cell lines were plated at ~ 80% confluency in medium containing lentiviral supernatant and 5 μg/ml polybrene, spinfected at 1800 rpm for 1 hr. in a pre-warmed plate in a Rotina 420R centrifuge (Hettich) at 32 °C, and subsequently left overnight in the incubator. If applicable, puromycin selection medium was added 24 hr. post-transduction. Cultures successfully transduced with fluorescent protein plasmids were sorted on the BD FACS ARIA™ II SORP Cell Sorter (BD Biosciences).

### CRISPR-based kinome essentiality screen

The kinome CRISPR sgRNA library was designed and generated at the Netherlands Cancer Institute (NKI) and contained 50 non-targeting sgRNAs, 50 sgRNAs targeting essential genes (COPB1, KPNB1, NUP98, PSMB2, PSMC4, PSMD6, PSMD11, RPL3, RPL11, RPS13) and 5860 sgRNAs targeting 502 kinases cloned into the LentiCRISPRv2.1 backbone through Gibson assembly. Plasmid library was expanded through electroporation into Endura™ electrocompetent cells (Cat. #60242-0, Lucigen). Fourteen cell lines were lentivirally transduced in triplicate with the CRISPR kinome library at a multiplicity of infection of ~ 0.3. Successfully transduced cells were selected using puromycin for 48 (HCT116, HuTu-80, KM12, MDST8, OUMS-23, RKO, SW48) or 72 hours (Colo320-HSR, HT55, LS180, LS513, LS1034, RCM-1, SW948) after which the T_0_ reference sample was collected. All lines were cultured twice before final harvest and collection of the T_1_ sample after 9 divisions. Division time of each line was reported on before [8]. DNA was extracted from T_0_ and T_1_ samples and sequencing library was prepared by PCR using Phusion™ high fidelity DNA polymerase (Cat. #F530L, ThermoFisher Scientific) and sgRNA abundance in each replicate was determined through Illumina deep sequencing.

Normalization and a differential analysis of T_1_ versus T_0_ on the sgRNA level was performed using DESeq2 [16]. Raw normalized sgRNA counts per sample per cell line are available in Supplementary Table S1. A differential analysis of T_1_ versus T_0_ on the gene level was performed using MAGeCK [17], which provides a False Discovery Rate (FDR) value. At the time of analysis, MAGeCK output did not include the median of the Log2FoldChange of the “good sgRNAs” (p.low ≤0.25) and it was therefore calculated separately based on the DESeq2 results. PAK2 was identified as a CMS4-specific drop-out by comparing the fold depletion of abundance of PAK2-targeting sgRNAs in T_1_ to T_0_ in CMS4 versus CMS1–3 cell lines, negative FDR threshold was set to ≤0.01.

### Data processing and gene expression normalization

Eleven publicly available datasets containing colorectal tumor samples from the Gene Expression Omnibus (GSE13067, GSE13294, GSE14333, GSE17536, GSE20916, GSE2109, GSE23878, GSE33113, GSE35896, GSE37892, and GSE39582) were downloaded and normalized using the rma function of the ‘affy’ package [18]. All these samples were combined with a private dataset (KFSYSCC) profiled on the same affymetrix platform (133plus2). Quantile normalization was performed on the combined samples prior to using the ComBat batch effect removal method [19].

RNAseq data for colorectal cancer tumor samples was obtained from the TCGA [2] dataset and normalized to the fpkm values. Normalized TCGA data and combined affymetrix datasets were aggregated into a single matrix of 2416 samples and the ComBat method was performed to further remove batch effects. All of these samples have been used in consensus molecular subtyping of colorectal cancer and CMS labels of samples were also defined during this work [4].

For the analysis of the cell line samples, datasets GSE36133, GSE100478, GSE59857 and GSE68950 containing profiles of established cancer cell lines and GSE100479 and GSE100549 containing profiles of primary cell lines, complemented with profiles of 9 spheroid cultures derived in in the laboratory of Prof. Dr. Giorgio Stassi in Palermo (unpublished data) were normalized and CMS stratified as previously described [7].

Heatmaps representative of the two top hits for each CMS subtype were generated using the pheatmap function of the ‘pheatmap’ package from CRAN.

### Competition assay

WT cells and stably transduced pTRIPZ shRNA non-silencing (NS) or PAK2-KD cell lines were treated for 6 days with 1 μg/ml doxycycline to induce turboRFP and linked shRNA expression before sort. Left and right sort gate for 20% turboRFP-high, shRNA-high cells were set to capture 20% highest turbo-RFP expressing PAK2-KD cells in population and copied to NS cell line. NS and PAK2-KD shRNA-high cells were sorted in two separate tubes, after which live WT cells were sorted on top into both tubes to reach a final mixture of 70% shRNA-high and 30% WT cells in each tube. Sorted, non-mixed WT cells were included throughout the experiment as turboRFP-negative controls. Part of the shRNA-high + WT mixtures was plated in triplicate and remainder was analyzed on FACS to determine exact shRNA-high:WT cell ratio on day 0 post-sort in NS + WT and PAK2-KD + WT co-culture conditions. 7-AAD was used to exclude dead cells. Plated triplicates were maintained in culture and then harvested, passaged and analyzed on FACS on days 7, 10, 14, 17, 21, 24 and 28 post-sort using the same FACS laser intensity and gate settings as on day 0. 7-AAD was again used to exclude dead cells. Contribution of shRNA-high cells to both mixed populations was first normalized to their contribution (%) measured at day 0 post-sort. Percentages plotted in Fig. 2E were obtained by subsequently setting NS shRNA contribution to 100% for each day and then normalizing PAK2-KD contribution to co-culture population to NS contribution.

### Generation of stable CRISPR PAK2 knock-out clones

sgRNA sequence targeting PAK2 was available via the LentiCRISPRv2.1 kinome library: 5′ – TATGCCACTCTCTTACCAGG. sgRNA was cloned into pSpCas9(BB)-2A-GFP (pX458) as described [20] and obtained plasmid was transformed into One Shot™ TOP10 Chemically Competent cells (Cat. # C404003, ThermoFisher Scientific). Successful insertion of sgRNA was confirmed with Sanger sequencing. CRC cell lines were plated in a 6 well plate at a density to reach ~ 70–80% confluency for transfection the next day with 2,5 μg plasmid and FuGENE® HD Transfection Reagent (Cat. #E2311, Promega) in a 1:3 ratio. Transfection mixture was left overnight after which cells were expanded to a T25. Successfully transfected GFP positive cells were single cell sorted into 96-well plates. Single cell clones that successfully grew out were harvested and part expanded, part plated in a new 96-well plate to obtain DNA lysates. When wells on 96-well plate were full, medium was aspired, cells were washed with PBS and subsequently left in 50 μl trypsin per well at 37 °C for 1 hr. to detach cells and partially disrupt membrane. Plates were frozen down, thawed and crude DNA lysates were collected in Eppendorf tubes to boil at 99 °C for 10 min. Two microliters of this lysate was transferred to a PCR reaction containing Platinum Taq buffer, Platinum Taq DNA polymerase, 50 mM MgCl_2_ (all Cat. #10966-034, Invitrogen), 10 mM dNTPs, forward (5′ – tggtgaaaccccgtctctac) and reverse (5′- ccgtttctggcaaacctatg) primers. The following conditions were set up for PCR: 10 min 94 °C, 35 cycles of 30 sec 94 °C, 30 sec 61 °C and 30 sec 72 °C, then final extension for 10 min 72 °C. Two microliters of PCR product was used in BigDye Terminator v3.1 (Cat. #4337455, Applied Biosystems) Sanger sequencing reaction using the reverse primer. Sequences of single cell clones were compared with WT PAK2 sequence using TIDE analysis [21] to identify full PAK2 knock-out clones.

### RNA isolation, cDNA conversion and qRT-PCR on cell line samples

Cell lines were grown to reach 70–80% confluency and were harvested using trypsin. Cell pellets were snap frozen in liquid nitrogen and stored at − 80 °C until RNA isolation. RNA was isolated using the NucleoSpin RNA kit according to manufacturer’s protocol (Cat. #740955, Machery-Nagel) and quantified on a NanoDrop 2000 (Thermo Fisher Scientific, Waltham, MA, USA). One microgram of RNA was converted into cDNA using the Superscript III reverse transcriptase kit according to manufacturer’s protocol (Cat. # 18080085, Invitrogen). qRT-PCR was performed using SYBR green I (Cat. #4887352001, Roche) on a LightCycler® 480 II machine (Roche). Primer sequences were obtained from PrimerBank [22] or designed in house and are listed in the table below. All obtained Ct values were normalized to the expression of *GUSB*, results normalized to *ATP5E* were similar.

**Table:** 

Gene	Forward	Reverse
*PAK1*	5′ - AGGGGAGTTTACGGGAATGC	5′ - TCTTCTGCTCCGACTTAGTGATA
*PAK2*	5′ - CTGAGCTTTACTCCTCCTGAGA	5′ - GGGTGCTTCTGTTCCCTTGG
*PAK3*	5′ - CCAGGCTTCGCTCTATCTTCC	5′ - TCAAACCCCACATGAATCGTATG
*ARHGEF7 (βPIX)*	5′ - GCCAGTATCGGAGTTTGGACA	5′ - TTGGGAGGGCTCTTCAGTGT
*ATP5E*	5′ - CCGCTTTCGCTACAGCAT	5′ - TGGGAGTATCGGATGTAGCTG
*GUSB*	5′ - TGGTTGGAGAGCTCATTTGGA	5′ - GCACTCTCGTCGGTGACTGTT

### Western blotting

Lysates of all cell lines were generated from cultures grown to 70–80% confluency. Cells were washed twice with ice cold PBS and lysed in 2x Laemmli buffer plus 10% β-mercaptoethanol and 1x Halt™ protease/phosphatase inhibitor cocktail (Cat. #78440, Thermo Fisher Scientific) and scraped from the culture dish. Lysates were collected in Eppendorf tubes, boiled for 10 min at 95 °C and centrifuged at 4 °C at maximum speed for 10 min to remove remaining cell debris. Protein concentration was determined using the Protein Quantification Assay (Cat. #740967, Macherey-Nagel) before loading 20 μg of protein onto 4–15% Mini-PROTEAN® TGX™ Precast Gels (Cat. #4561086 and #4561036, Bio-Rad laboratories). Before proceeding with blotting for PAK1 and PAK2 expression in the whole cell lysate of WT CMS2 and CMS4 Sanger cell lines, the polyacrylamide gel was incubated for 15 min in electrophoresis buffer containing 1% 2,2,2-Trichloroethanol (Cat. #T54801, Sigma-Aldrich) to allow for tryptophan visualization and thereby comparison of amount of protein loaded between cell lines [23]. Gel was imaged using the UV sample tray of a Gel Dox EZ system (Bio-Rad Laboratories).

Protein was transferred using the Trans-Blot® Turbo™ RTA Mini PVDF Transfer Kit (Cat. #1704272, Bio-Rad Laboratories). Membranes were blocked in 5% bovine serum albumin in TBS + 0,1% Tween-20 (Sigma-Aldrich) for 1 hr. and subsequently probed overnight at 4 °C with primary antibody diluted in blocking buffer under light agitation.

Primary antibodies used: PAK1, 1:1000, Cat. #2602; PAK2, 1:1000, Cat. #2608; phospho-Cofilin (Ser3), 1:2000, Cat. #3313S; Cofilin, 1:5000, Cat. #5175S (all Cell Signaling Technology) and GAPDH, 1:5000, Cat. #MAB374/6C5 (Merck/Millipore). The following secondary antibodies were diluted in 5% bovine serum albumin in TBS + 0,1% Tween-20: goat-α-rabbit-IgG-HRP, 1:5000, Cat. #7074 (Cell Signaling Technologies) and goat-α-mouse-IgG-HRP, 1:5000, cat. #1031-05 (SouthernBiotech, Birmingham, AL, USA). Blots were imaged using Lumi-Light^PLUS^ western blotting substrate (Cat. #12015196001, Roche) on an ImageQuant™ LAS 4000 machine (GE Healthcare, Chicago, IL, USA). All in between washing steps were performed with TBS + 0,1% Tween-20. Quantification of Western blot images was performed as previously described [24].

### Flow cytometry

Cells were harvested with trypsin and made single cell prior to FACS assays. All sorting experiments using FACS were performed on a BD FACS ARIA™ II SORP Cell Sorter (BD Biosciences). All FACS analysis experiments were performed on a BD FACSCanto™ II (BD Biosciences). Unstained or untransduced wildtype (WT) cells functioned as negative controls. 7-AAD (Cat. #559925, BD Biosciences) at a 1:100 dilution was added to samples to exclude dead cells. FACSDiva v8 software was used to acquire data, data analysis was performed with FlowJo software.

For the Nicoletti assay, 190,000 (HuTu-80) and 125,000 (MDST8) cells were plated per well of a 6 well plate. Medium was collected and combined with cells harvested using trypsin 48 hr. later. Cells were pelleted, taken up in freshly prepared Nicoletti buffer (50 μg/ml propidium iodide, 0.1% sodium citrate and 0.1% Triton X-100 in ddH_2_O) and left at 4 °C overnight before analysis on BD FACS Canto II.

### Cell viability assays

The proliferative capacity of PAK2 knock-out clones compared to wildtype cell lines was performed by seeding cells in a 96 well plate at a density of 80 cells per well in 100 μL medium on plastic (Fig. 3B; Supplementary Fig. S2C) or at 80 cells per well in 6 μL Matrigel (Corning) drops in 100 μL medium (Fig. 3D; Supplementary Fig. S2D). All experiments were performed in 12 technical replicates and 3 biological replicates. Four hour after plating, a baseline CellTiter Glo (Cat. #G7571, Promega) measurement was included (day 0), the remaining plates were measured 4 days and 7 days after plating. All measurements were performed according to the manufacturer’s protocol.

The effect of FRAX597 on the HuTu-80 and MDST8 cell lines as well as the p11t and p26t human organoids in Matrigel was assessed by plating 300 (HuTu-80), 150 (MDST8) or 1500 (p11t and p6t) cells per well in 6 μL Matrigel (Corning) drops in 100 μL medium, DMSO control, or medium containing increasing concentrations of FRAX597 (Cat. #S7271, SelleckChem, München, Germany). All experiments were performed in 6 technical replicates and 3 biological replicates. Four hours after plating, a baseline CellTiter Glo (Cat. #G7571, Promega) measurement was included (day 0), the remaining plates were measured 4 days after plating. All measurements were performed according to the manufacturer’s protocol.

The FRAX597 sensitivity assay was executed by seeding cells in 100 μL medium in a 96 well cell tissue culture plate and treating cells in triplicate with DMSO or a titration of FRAX597. The drug titration was printed using a HP D300e Digital Dispenser (Hewlett-Packard, Palo Alto, CA, USA). The number of cells plated per well were 4000 for HT55 and 1000 for HuTu-80 and MDST8. Cell proliferation was measured using the IncuCyte S3 (Sartorius, Göttingen, Germany). Images were acquired every 6 hours for 72 hours starting 1 hour after plating. Data analysis and quantification was performed using the IncuCyte Base Analysis Software. Experiments were performed in 3 technical replicates and 3 biological replicates.

The chemotherapy sensitivity assay was performed by seeding 80 cells per well in 100 μL medium in a 96 well cell tissue culture plate and treating cells in triplicate with DMSO or a titration of Oxaliplatin, 5-FU or Paclitaxel. The drug titration was printed using a HP D300e Digital Dispenser (Hewlett-Packard, Palo Alto, CA, USA). Cell viability was measured 4 hours after plating, as a day 0 baseline measurement, and 3 days after treatment using CellTiter Glo (Cat. #G7571, Promega) according to the manufacturer’s protocol. All experiments were performed in 3 technical replicates and 3 biological replicates.

### Immunohistochemistry

Per animal, three cross-sections of separate areas from the liver and one cross-section from the lungs were derived from FFPE material obtained in the mesenteric vein injection experiments. Sections were stained with a human-specific Ku80 antibody (C48E7, cat. #2180, Cell Signaling) and counterstained with hematoxylin by the NKI animal pathology department to accurately detect micro- and macro-metastases. Slides were scanned on an Aperio AT2 slide scanner (Leica). Metastatic burden was quantified in ImageJ as follows; first channels were separated by Color Deconvolution (vectors = [H&E DAB]), next the total tissue area was determined on channel 1 (hematoxylin) after application of a Gaussian Blur and AutoThreshold, lastly the number and area of all individual foci was measured on channel 3 (DAB) after application of a Gaussian Blur and AutoThreshold using the Analyze Particles function.

### Clonogenic assay

The clonogenic assay for βPIX targeting shRNA transduced cells was quantified using crystal violet as previously described [25]. Briefly, following lentiviral transduction with pLKO.1 non-silencing and βPIX targeting shRNAs and puromycin selection, an equal number of cells were plated for each condition in a 24 well plate in triplicate. After 48 hours cells were washed twice with PBS and a mixture of 500 μL 6.0% glutaraldehyde and 0.5% crystal violet was added per well. Cells were incubated at RT for 30 min after which the solution was removed. The plates were rinsed with tap water and left to dry overnight. Visualization of the plates was obtained with an HP Scanjet 4850 scanner. Quantification was performed by dissolving the crystal violet stained cells in 300 μL PBS containing 1% sodium dodecyl sulfate (SDS) per well for 1 hr. at RT on a shaker. The absorbance was then read on a SynergyTM HT multi-detection microplate reader (BioTek Instruments) at 570 nm wavelength.

### Immunofluorescence

An equal number of cells per condition were plated on top of glass coverslips in a 12 well plate. After 24 or 48 hr. of incubation, the medium was removed and cells were washed with PBS twice followed by fixation with 4% PFA for 10 min. The PFA was then removed and the cells were washed twice with PBS. Before staining, cells were permeabilized for 10 min using 1% Triton X-100 (Thermo Fischer) diluted in PBS. Next, samples were blocked using 5% Normal Goat Serum (Cat. # ab7481, Abcam) and 0,1% Triton X-100 in PBS for 30 min. Primary antibodies were diluted in BrightDiluent green antibody diluent (Cat. #UD09–999, Immunologic) according to manufacturer’s protocol and incubated overnight at 4 °C. The samples were washed twice with PBS and incubated in secondary antibody for 1 hr. at RT covered. Next, Hoechst-33,341 (Cat. #62249, Thermo Fischer) was used as a nuclear counterstain and incubated at 10 μg/ml in PBS for 5 min at RT. For the actin staining, samples were incubated after the permeabilization step with ActinRed™ 555 ReadyProbes™ Reagent (R37112, Thermo Fischer) according to manufacturer’s protocol, Hoechst-33,341 was also used as nuclear counterstain for these slides. The coverslips were then mounted with Prolong Gold antifade reagent (Cat. #P10144, Invitrogen) on glass slides. All stainings were analyzed with the SPSX8 confocal microscope (Leica) and stored at 4 °C. The following antibodies were used: anti-human vimentin (1:300; sc-73,259, Santa Cruz), anti-human β-tubulin (1:200; T7816/SAP.4G5, Sigma), anti-mouse Alexa Fluor 488 (1:500, A11029, Invitrogen).

### TIRF Lifeact/paxillin imaging and analysis

Lifeact-GFP and Paxillin-mCherry transduced MDST8 and HuTu-80 WT and PAK2-KO cells were plated on Lab-Tek chambered 1.0 borosilicated coverglass slides and cultured in SILAC DMEM Flex Medium (Cat. #A2493901, ThermoFischer) supplemented with 8% FBS, 1% D-glucose solution plus), 1 mM sodium, 0,5% glutamine solution and 50 units/mL of penicillin/streptomycin prior to imaging. For TIRF microscopy, an inverted NIKON Eclipse TI equipped with a 60 × 1.49 NA Apo TIRF (oil) objective, perfect focus system, Argon Ion Laser 457–514 nm (Melles Griot), Orange Diode Solid State Laser 594 nm 30 mW (Excelsior, Spectra-physics), dual band 488/594 nm TIRF filter cube (Chroma TRF59905 ET), and an Andor Zyla 4.2 plus sCMOS camera (without binning) was used. An Okolab cage incubator and humidified CO2 gas chamber set to 37 °C and 5% CO2 were used during the imaging process, as described before [26]. Image acquisition was performed every 30 seconds for 1–3 hr. total.

### Invasion and migration assay

In vitro cell invasion of MDST8 and HuTu-80 WT or PAK2-KO cell lines was measured using the Corning BioCoat Matrigel Invasion Chamber assay (Cat. #354480, Corning) with 8.0 μm PET membrane, at 48 hr. post seeding. In vitro cell migration of primary spheroid lines was measured using the Corning 8.0 μm Pore Polycarbonate Membrane inserts (Cat. #3422, Corning). An equal amount of cells for each condition was plated in serum-free medium (DMEM/F12 or RPMI) in the upper chambers. Culture medium containing 10% FCS was used in the lower chamber as a chemoattractant. After incubation for 48 hr. for the cell lines and 72 hr. for the primary lines at 37 °C, the medium was removed from the upper and lower chambers. The cells were fixed for 10 min with 4% paraformaldehyde, rinsed with PBS and the non-migrated cells plus coating were removed from the upper chamber. The migrated cells were stained using crystal violet solution (see above) for 30 min and then rinsed with tap water. The stained migrated cells were imaged on a Leica DM6 fluorescence microscope. Captured images were analyzed and quantified using Image J (ImageJ v1.50i, National institute of Health) and MATLAB software Briefly, crystal violet stained cells were identified as connected areas of positive pixels (intensity above manually chosen threshold) of a minimum size, to exclude background noise from filter pores or debris. Amount of crystal violet positive cells in this thresholded image were quantified in MATLAB for WT, NS, PAK2-KD and PAK2-KO images as the ratio of crystal violet positive cells on the total membrane area.

### In vivo experiments

All mouse experiments were approved by animal experimentation committees at the respective institutes and performed according to institutional and national guidelines. Experiments were carried out at the following institutes, under the listed nationally registered license numbers: (i) subcutaneous injections, University of Palermo, D.L. number 26 March 4th 2014, 154/2017-PR; (ii) intraperitoneal injections, Academic Medical Center (AMC), AVD118002016493; (iii) mesenteric vein injections, Netherlands Cancer Institute (NKI), AVD3010020172464. All mice were housed in temperature-controlled facilities (20–24 °C) at 40–70% humidity with a 12 h dark/light cycle and ad libitum access to food and water. Animals were sacrificed in accordance with Directive 2010/63/EU guidelines (D.lgs 26/2014). Mice were randomly assigned to experimental groups, experiments and analysis were not blinded, except for quantification of metastatic liver colonization and secondary spread to the lungs for the mesenteric vein injection experiments. Sample sizes were determined based on previous studies with similar experimental designs [8, 11, 27].

Six week old male NOD-SCID mice were purchased from Charles River Laboratories and subcutaneously injected with 1•10^6^ viable cancer cells suspended in 100 μL 2% BSA in PBS before reaching 12 weeks of age. Mice were monitored and weighed and tumor size was measured using a digital caliper twice a week. Tumor volume was calculated using the formula (smallest diameter)^2^ • largest diameter • π/6. Mice were sacrificed once one of the following endpoints was met: (i) tumor volume of ≥2 cm^3^ or (ii) humane endpoint, mouse suffering as indicated by eg. weight loss or impaired movement of hind leg due to position of the tumor.

The experimental set-up to assess peritoneal metastatic potential of CRC cell lines was extensively described before [11]. Female Hsd:Athymic Nude-*Foxn1*^*nu*^ mice (6–12 weeks old, Envigo) were injected intraperitoneally with 1•10^4^ CRC cells suspended in a cold 50% Matrigel – 50% culture medium solution of 100 μl volume. Mice were monitored and weighed twice a week and sacrificed 6 (MDST8) or 7 weeks (HuTu-80) post-injection to derive the peritoneal cancer index (PCI). No severe discomfort (eg. > 15% weight loss within 2 days) was observed during these studies.

The mesenteric vein injection model was described before [27]. Eight to fourteen weeks old male NOD.Cg-Prkdc^scid^ Il2rg^tm1Wjl^/SzJ (NSG, Cat. #005557, The Jackson Laboratory) mice were injected with 5•10^4^ WT HuTu-80-Dendra2 cells, PAK2-KO HuTu-80-LeGO-tdTomato cells or a 50:50 mixture of these two lines for a total of 5•10^4^ cells suspended in 100 μl PBS. Six mice were used for the WT condition, five for the other two. One mouse dropped out prematurely in the WT and WT-KO mixed condition, shortly after surgery. One mouse was mis-injected in both the WT and KO group. All 4 remaining animals in each group were sacrificed 28 days post-injection. Livers and lungs were excised and fluorescent Dendra2 and tdTomato signal was imaged macroscopically on an AxioZoom.V16 (Zeiss), using a Plan-neofluar Z 1.0x/0.25 FWD 56 mm objective and a Monochome Axiocam 503 camera. Pieces of livers and lungs were formalin-fixed, paraffin-embedded (FFPE) and snap frozen for further microscopic quantification of tumor burden.

## Results

### A CRISPR-CAS9 drop-out screen reveals unique essential kinases for the CMS of CRC

**Fig. 1:**
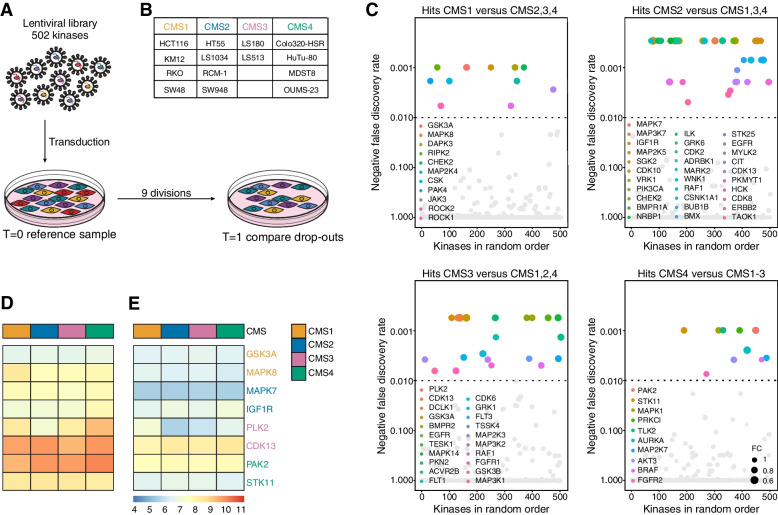
A CRISPR-based dropout screen identifies sensitivities for each of the CMS of CRC. **A**. Schematic overview of set-up CRISPR-based drop-out screen. **B**. Table of the cell lines used in the drop-out screen representing each of the CMS. **C**. Bubble plots represent outcome of MAGeCK analysis comparing, clockwise, CMS1 to CMS2–4, CMS2 to CMS1, 3, 4, CMS3 to CMS1, 2, 4 and CMS4 to CMS1–3 CRC cell lines. Each bubble represents one kinase, listed randomly. All significant CMS-specific drop-outs are highlighted in color. Negative false discovery rate for MAGeCK output was set to 0.01. Bubble size represents calculated median fold change for all sgRNAs targeting the same gene. FC = Fold change. **D**, **E**. Heatmaps depicting Z-score transformed 2Log gene expression of the top 2 most significant drop-outs for each CMS in cell lines (**D**) and CRC tumor samples (**E**). The color of the labeled kinases indicates the subtype for which it significantly drops-out, as is depicted in the legend

To identify subtype-specific vulnerabilities, a CRISPR-Cas9-based drop-out screen, specifically targeting the kinome was performed. This set-up was aimed at identifying which kinases are essential for survival or proliferation of a cell line by tracing the abundance of a given guide RNA (gRNA) in the population over the course of 9 cell divisions (Fig. 1A). To discriminate between subtypes, a defined set of 14 CRC cell lines representing each CMS and harboring various CRC driver mutations was used (Fig. 1B). The focus on the kinome was inspired by the fact that a wide selection of small molecules is available to inhibit this class of proteins, thereby enhancing the chances of uncovering clinically actionable targets. The analysis revealed that knock-out of several kinases resulted in a general drop-out in all lines and these related mostly to regulation of cell cycle, transcription or translation, such as CHEK1, CDK1, WEE1 and PLK1 (Supplementary Table S2). Subsequently, cell lines were grouped using the CMS annotation of all 14 lines and a comparison was made between the subtypes. Intriguingly, all CMSs contained essential kinases that dropped-out in a subtype specific manner (Fig. 1C). A relatively large number of kinases proved to be essential for survival and/or growth of the epithelial subtypes CMS2 and CMS3. This subtype selectivity did not appear to directly relate to CMS-specific expression of the kinases as analysis of mRNA expression levels of the top 2 kinase drop-outs per CMS showed a relatively stable expression across the subtypes both in cell lines (Fig. 1D) and tumor samples (Fig. 1E). This suggested that subtype-specific differences in sensitivity were the result of differential usage of the kinases and confirmed that the subtypes do display biologically distinct wiring.

### CMS4 cancer cells are uniquely sensitive to PAK2 loss

The mesenchymal subtype CMS4 is characterized by therapy resistance as well as a more aggressive nature with a higher propensity to metastasize [4]. To validate the findings of the drop-out screen and to identify pathways crucial for the aggressive CMS4 cancers, drop-outs for the CMS4 cell lines were chosen for further study. Ten kinases with a significantly higher drop-out rate in CMS4 were identified (Fig. 1C). Among these is AKT3, which we recently identified as a driver of CMS4 outgrowth following identification by a bioinformatical pipeline [24]. Closer analysis of these 10 hits on the individual cell line level singled out PAK2 as the lead hit for CMS4 (Supplementary Table S2). The sensitivity to loss of PAK2 was, unlike the other 9 candidates, unique to CMS4 cell lines and evident in all but one of the CMS4 lines included in the screen.

**Fig. 2:**
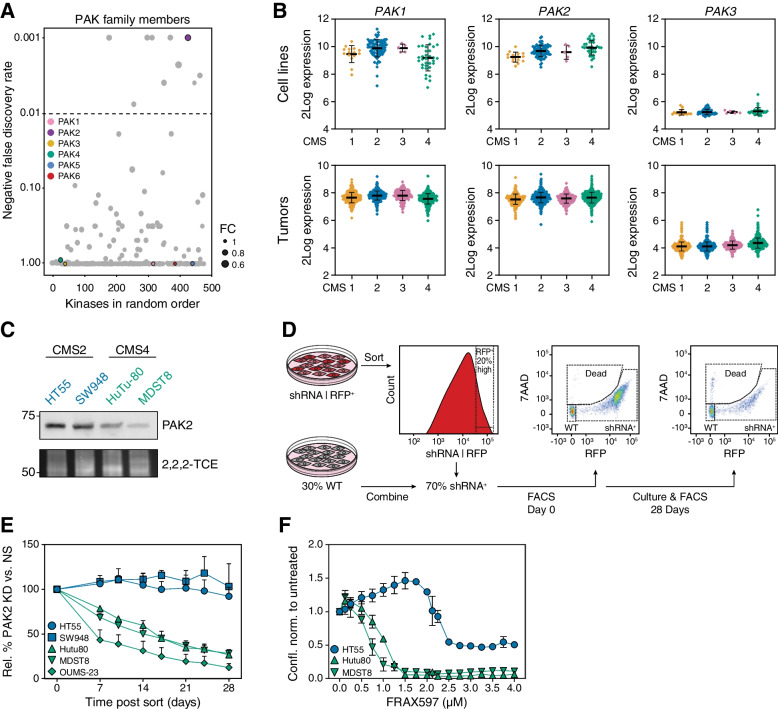
PAK2 is a CMS4-specific vulnerability. **A**. Bubble plot representing outcome of MAGeCK analysis comparing PAK family members in CMS4 to CMS1–3 CRC cell lines. Each bubble represents one kinase, listed randomly. Negative false discovery rate for MAGeCK output was set to 0.01. Bubble size represents calculated median fold change for all sgRNAs targeting the same gene. FC = Fold change. **B**. 2Log mRNA expression levels for PAK family I members in CRC (primary) cell lines (upper panels) and tumors (lower panels) as determined by microarray or RNA sequencing. Bars indicate mean, error bars represent standard deviation (S.D). **C**. Western blot for PAK2 protein expression in HT55 & SW948 (CMS2) and HuTu-80 & MDST8 (CMS4). 2,2,2-Trichloroethanol (2,2,2TCE) signal (excerpt taken around 60 kDa region) indicates amount of protein loaded per cell line. Numbers on the left represent molecular weight in kDa according to a stained protein ladder loaded in the gel. Representative blot of *N* = 3 is displayed. **D**. Schematic overview of FACS-based co-culture/competition assay in which WT cells were mixed with either doxycycline-induced non-silencing control shRNA or PAK2-targeting shRNA cells at a 30%:70% ratio, respectively. **E**. Relative contribution of PAK2-KD cells to co-culture in competition assay set-up, over time. Contribution of shRNA-turboRFP expressing cells to population was first normalized to contribution on day 0 post sort, and subsequently calculated relative to contribution of NS shRNA-turboRFP expressing cells at each individual time-point. CMS2 cell lines indicated in blue, CMS4 in green. *N* = 3, mean and standard error of means (s.e.m., error bars) are plotted. **F**. Cell confluence measured over time with IncuCyte live-cell imaging, up to 3 days after start of treatment. Values were normalized to the DMSO treated control. CMS2 indicated in blue, CMS4 in green. Representative experiment of *N* = 3 is displayed, mean and standard error of means (s.e.m., error bars) are plotted

PAK2 is a member of the group I PAK family (comprised of PAK1–3), and closely related to the group II PAK family members, PAK4–6 [28]. PAK2 serves a role downstream of Rho GTPases RAC and CDC42 and is involved in actin cytoskeleton remodeling, but also has a reported role in centrosome activity and membrane blebbing during apoptosis [29, 30]. Intriguingly, no sensitivity to loss of any of the other PAK family members was observed in CMS4 lines (Fig. 2A), neither to PAK2’s distant relatives PAK4–6, nor to its closer relatives PAK1 and PAK3, further highlighting the unique role of PAK2 in the mesenchymal CRC subtype. Analysis of PAK family gene expression in tumors and cell lines showed that this subtype-specific sensitivity to loss of PAK2 could not be attributed to differential expression of other PAK family members (Fig. 2B; Supplementary Fig. S1A-D). To understand the dependency of mesenchymal CRC cell lines on PAK2, a direct comparison was made between the two most distant subtypes, being the epithelial subgroup CMS2 and the mesenchymal subgroup CMS4. First, analysis of protein expression levels confirmed the observation on the mRNA level and indicated that PAK2 sensitivity was not linked to overabundant PAK2 expression in CMS4 lines used in the screen (Fig. 2C). Second, an independent validation of the CMS4 dependency on PAK2 was performed using shRNA mediated knockdown in a competition assay in which wildtype (WT) cells were co-cultured with PAK2 knockdown cells (PAK2-KD) and the contribution of knockdown cells to the total population was followed over time (schematic overview in Fig. 2D). Effective knockdown of *PAK2* was observed in all doxycycline-induced turboRFP^+^-shRNA^+^ cell lines (Supplementary Fig. S1E), while off-target or compensatory expression changes in *PAK1* were negligible across cell lines (Supplementary Fig. S1F). The competition assay confirmed that PAK2-KD cells were specifically lost from the population in CMS4 cell lines in a time-dependent fashion, whereas growth of CMS2 PAK2-KD cell lines was unaffected (Fig. 2E). PAK2 sensitivity was extended to two additional CMS4 cell lines not included in the original CRISPR-Cas9 drop-out screen, pointing to a more general dependency of mesenchymal lines on this kinase (Supplementary Fig. S1G, H). Importantly, use of a PAK1/2/3 inhibitor, FRAX597, indicated that CMS4 lines were clearly more sensitive to inhibition of this family of kinases compared to CMS2 lines, suggesting that the PAK2 dependency also translates into sensitivity towards a chemical inhibitor (Fig. 2F).

### PAK2 is essential for CMS4 tumor growth

**Fig. 3:**
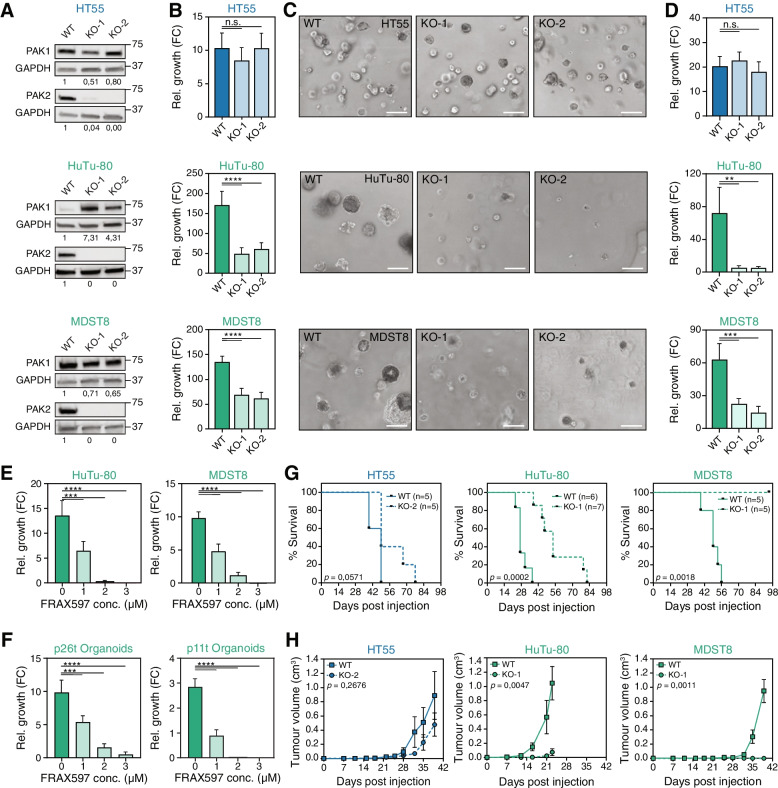
Loss of PAK2 leads to reduced outgrowth of CMS4 cell lines in vitro and in vivo. **A**. Western blots to assess PAK1 and PAK2 protein levels in CRISPR-Cas9-editted single cell clones. Images representative of *N* = 3. WT = Wildtype, KO = knock-out. Numbers on the right represent molecular weight in kDa according to a stained protein ladder loaded in the gel. **B**. Outgrowth of WT and PAK2-KO cells plated on plastic relative to day 0, as measured 7 days post-plating by CellTiter Glo. Representative experiment of *N* = 3 is shown. Data plotted is mean ± S.D. N.S. = not significant, **** = *P* < 0.0001 in two-tailed, Welch-corrected Student’s T-test. **C**. Representative phase contrast images of WT and PAK2-KO cells grown in Matrigel droplets for 7 days. Scale bars represent 250 μm. **D**. Quantification of outgrowth in Matrigel 7 days post-plating, relative to day 0, as measured with CellTiter Glo. FC = fold change relative to day 0 measurement. Representative experiment of *N* = 3 is shown. Data plotted is mean ± S.D. N.S. = not significant, ** = *P* < 0.005, *** *P* < 0.001 in two-tailed Welch-corrected Student’s T-test. **E, F**. Average relative growth of cells (**E**) or human organoids (**F**) after 4 days of treatment with FRAX597 at different concentrations or DMSO treated control (0) measured using CellTiter Glo. Representative experiment of *N* = 3 is shown. Data plotted is mean ± S.D.**** = *P* < 0.0001, *** *P* < 0.001 in two-tailed, Welch-corrected Student’s T-test. **G**. Kaplan-Meier survival curves of mice injected subcutaneously with WT or PAK2-KO cells. Number of mice per group indicated in graph. *P*-value calculated using log-rank Mantel-Cox test. **H**. Tumor volume as measured over time. Mean ± s.e.m. is plotted, data shown until the time-point at which ≥1 animal in a group was sacrificed. Throughout the figure CMS2 is indicated in blue, CMS4 in green. *P*-values calculated with a 2way ANOVA statistical test

To mimic complete loss of PAK2, as achieved in the drop-out screen, PAK2 CRISPR-Cas9 knock-out (KO) clones were generated for HT55 (CMS2) and HuTu-80, MDST8 and OUMS-23 (CMS4) (Fig. 3A; Supplementary Fig. S2A). In line with the drop-out screen and shRNA validation, in vitro outgrowth was impaired in CMS4 lines upon loss of PAK2, perhaps best exemplified by the observation that OUMS-23 PAK2 KO cultures crashed rapidly and could not be maintained in culture (Supplementary Fig. S2B). The other two CMS4 lines displayed reduced outgrowth in 2D cultures (Fig. 3B; Supplementary Fig. S2C) and even stronger impact in Matrigel-based 3D culture conditions (Fig. 3C, D; Supplementary Fig. S2D), which reportedly better mimics the in vivo setting. Vice versa, CMS2 cells were not affected in their outgrowth when deficient for PAK2 (Fig. 3B-D; Supplementary Fig. S2C, D). It has been previously reported that CMS2 and CMS4 cell lines are differentially sensitive to chemotherapy [8]. To explore if loss of PAK2 would result in increased sensitivity to commonly used chemotherapy in CRC, CMS2 and CMS4 WT and PAK2 KO cultures were treated with 5-fluoruracil (5-FU) and Oxaliplatin, Deletion of PAK2 did not result in significantly increased response efficiency for either of the drugs, neither in CMS2 nor in CMS4 lines (Supplementary Fig. S2E, F). The effect of Paclitaxel, a microtubule stabilizing drug [31], was also tested in WT and PAK2 KO cell lines. It was hypothesized that since PAK2 is involved in actin cytoskeleton remodeling and apoptosis [29, 30], this would result in increased sensitivity to Paclitaxel in combination with loss of PAK2. Similar to the response to 5-FU and Oxaliplatin, PAK2 KO cell lines did not show increased sensitivity to Paclitaxel (Supplementary Fig. S2G). Next, the effect of the PAK1/2/3 inhibitor FRAX597 was also measured in CMS4 cells in the 3D culture setting to further study its potential as a targeted treatment for this subtype. Both HuTu-80 and MDST8 were highly sensitive to FRAX597 at low doses in this setting (Fig. 3E). Importantly, CMS4 organoids derived from patient tumor samples were likewise strongly affected by PAK inhibition, indicating that this is a general CMS4 dependency (Fig. 3F). This dependency was not only evident in vitro but extended to in vivo tumor growth upon subcutaneous injection, which was completely impaired for MDST8 and significantly delayed for HuTu-80. In contrast, the epithelial CMS2 line HT55 was not dependent on PAK2 for subcutaneous tumor growth (Fig. 3G, H), emphasizing the relevance of our findings for tumor growth in vivo.

### PAK2 affects actin cytoskeletal remodeling in CMS4 cell lines

**Fig. 4:**
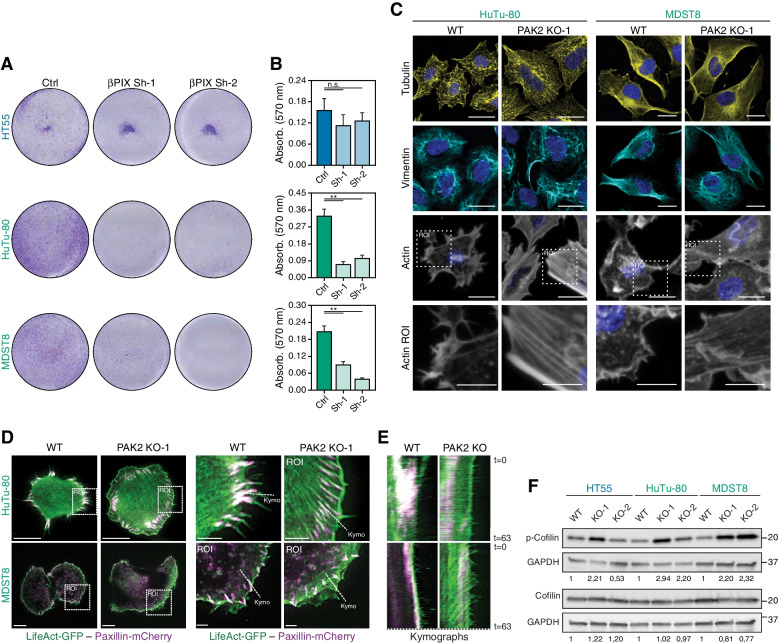
PAK2 is essential for attachment signaling and orchestrates actin cytoskeleton remodeling and focal adhesion localization. **A**, **B**. Representative images (**A**) and quantification (**B**) of crystal violet-stained clonogenic assays with cells expressing non-silencing or *ARHGEF7/βPIX* targeting shRNAs. *N* = 3, mean ± standard deviation are plotted, N.S. = not significant, ** = *P* < 0.01 as calculated in two-tailed, Welch-corrected Student’s T-test. **C**. Stainings for tubulin, vimentin and actin (phalloidin) 24 hours post-plating. Region of interest (ROI) magnified for actin staining indicated in white dashed box. Scale bar represents 20 μm, ROI images scale bar represents 10 μm. **D**, **E**. Stills from time-lapse TIRF microscopy at t = 0 of cell lines transduced with Lifeact-GFP (green) and paxillin-mCherry (magenta). Region of interest (ROI) selected is outlined by white dashed box (**D**). White dashed line in ROI images indicates region selected for kymographs (**E**). Scale bar left images represent 20 μm, ROI images 5 μm. See corresponding movies “Supplementary Movies 1, 2, 3, 4, 5 and 6”, available online for the full time-lapse recording. **F**. Representative western blot images showing phosphorylated cofilin (Ser3) and total cofilin in PAK2 proficient (WT) and deficient (KO) cells. Numbers on the right represent molecular weight in kDa according to a stained protein ladder loaded in the gel. Throughout the figure CMS2 is indicated in blue, CMS4 in green

Next the mechanism underlying the selective dependency of CMS4 on PAK2 was investigated. Previously, it was reported that caspase-cleaved active PAK2 is involved in the onset of apoptotic blebbing during apoptosis, while full length PAK2 was shown to prevent apoptosis by phosphorylating BAD and inhibiting caspase 3/7 activation [32–34]. Despite this role in regulating apoptosis, no overt difference in apoptotic rates were observed for the PAK2 KO cells, suggesting that cell death variation did not significantly contribute to the differences in growth rate (Supplementary Fig. S2H). A more concrete lead for the impact of PAK2 loss was evident from the bright field images of the PAK2 KO lines, which showed significant changes in the cellular shape with a more flattened morphology as compared to the wildtype cells (Supplementary Fig. S2I). This pointed to a role for PAK2 in cellular shape and potentially cytoskeletal rearrangements in CMS4 lines. Coinciding with this notion, inspection of a publicly available database of large CRISPR-based genetic screens (DEPMAP/PICKLES [35, 36]) unveiled a clear association between sensitivity to loss of PAK2 and loss of its binding partner βPIX (ARHGEF7) across a large panel of pan-cancer cell lines. This interaction between βPIX and PAK2 is reported to regulate actin cytoskeleton remodeling, focal adhesion (FA) disassembly and as a consequence migration [37, 38]. To determine whether the selective dependency of CMS4 cell lines would extend to βPIX, its expression was knocked down in CMS4 and CMS2 lines. Strikingly, βPIX knockdown also strongly reduced outgrowth of CMS4 cell lines, while the CMS2 line HT55 was insensitive to reduced βPIX expression (Fig. 4A, B; Supplementary Fig. S3A). This again confirmed the unique requirement of mesenchymal CRC lines for a βPIX/PAK2-dependent pathway and suggested that scaffolding of PAK2 at the membrane by βPIX is important in CMS4 outgrowth. Direct evidence for a role in cytoskeletal remodeling came from stainings for different filament networks. Both microfilaments (vimentin) and microtubules (tubulin) were comparable between PAK2 proficient and deficient cells. In contrast, intermediate filaments (actin) were significantly changed as indicated by a reduced amount of filopodia, increased deposition of actin at the membrane and an apparent increase in stress fibers in the PAK2 KO lines (Fig. 4C). To determine the dynamics of actin cytoskeleton remodeling and FA location in the absence of PAK2, CMS4 lines were transduced with LifeAct-GFP and Paxillin-mCherry and used for total internal reflection fluorescence microscopy (TIRF) imaging. Consistent with the phalloidin staining, TIRF imaging showed that PAK2 KO cells contained significant amounts of stress fibers with actin fibers concentrated at the cell membrane. The real-time analysis also captured the striking changes in filopodia dynamics with protrusions appearing at high frequency with a rapid turnover in WT cells, whereas they were strongly reduced and less abundant with slower turnover in PAK2-KO cells. Concurrently, lamellipodia were more evidently detected in the KO cells. The inclusion of paxillin-mCherry during filming revealed that the differences in actin distribution and dynamics at the membrane was related to the localization of FA in WT and PAK2 KO cells. FA were concentrated at the very edge of the membrane in WT cells connected to filopodia formation, whereas actin protruded well beyond the FA positions in PAK2 KO cells forming lamellipodia (Fig. 4D, E; Supplementary Fig. S3B-E; Supplementary Movies S1, S2, S3, S4, S5 and S6, available online).

Active cytoskeletal remodeling is regulated by an interplay between actin severing and depolymerization controlled by cofilin and filament extension via profilin. Cofilin activity is indirectly controlled by PAK1 and PAK2 via LIM kinase (LIMK) through phosphorylation [39]. Strikingly, phosphorylated cofilin levels were significantly increased in PAK2-KO cells as compared to WT cells (Fig. 4F), which correlates with the cytoskeletal phenotype observed: a less dynamic actin cytoskeleton with reduced filopodia formation, a shift in the localization of FA as well as the emergence of lamellipodia and stress fibers. Taken together, these differences illustrate that CMS4 cell lines require PAK2 to orchestrate actin cytoskeleton remodeling for the rapid emergence and turnover of filopodia, which are key structures for cancer cell invasion [40].

### PAK2 deficient CMS4 cell lines lack invasive and metastatic capacity

**Fig. 5:**
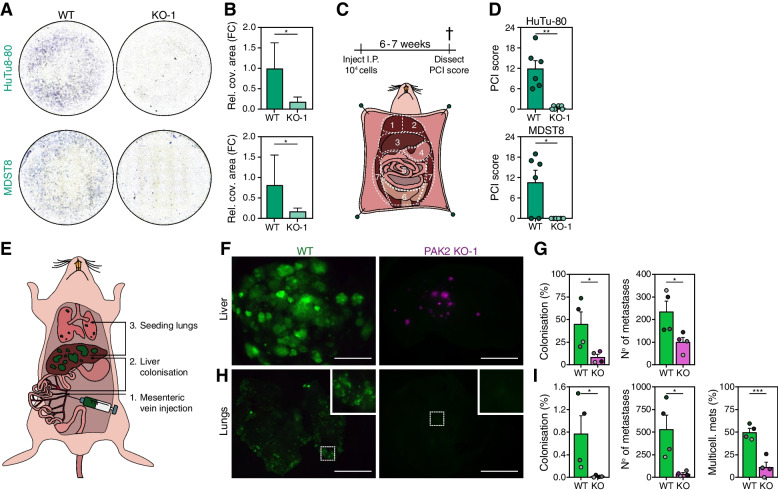
CMS4 cell lines depend on PAK2 for invasion and metastasis. **A**, **B**. Representative images (**A**) and quantification (**B**) of Matrigel-coated Transwell experiments. Bar plots display relative area covered by cells (purple) in Transwell image. FC = fold change relative to WT mean value. Mean + S.D. derived from *N* = 5 are plotted. * = *P* < 0.05 in one-tailed Mann-Whitney test. **C**. Illustration indicates regions scored for abundance of metastatic foci in the intraperitoneal metastasis mouse model. Maximum score per region is 3 and thus 21 in total. **D**. Bar graphs depict mean PCI score + s.e.m. Individual dots indicate score for one mouse. ** = *P* < 0.005, * = *P* < 0.05. **E**. Schematic drawing of mesenteric vein injection assessing capacity to colonize liver and secondary spread to lungs. **F-I**. Macroscopic images (**F, H**) of tumor burden in full liver and lungs taken directly after sacrificing mice injected with HuTu-80 WT-Dendra (green) and PAK2-KO-tdTomato (magenta) cells. Colonization of organs and number of metastases was quantified through immunohistochemistry for human Ku80 on three liver sections and 1 lung section per mouse (**G, I**). Bar graphs indicate mean + s.e.m. * = *P* < 0.05, *** = *P* < 0.001 as calculated by one-tailed, unpaired Student’s T-test. Scale bar represents 500 *μm*. Each colored bubble indicated on **G, I** represents an individual mouse used in the experiment

The observation that CMS4 cell lines depended on both PAK2 and βPIX in combination with the clear impact on actin cytoskeleton dynamics, suggested that the reduction in outgrowth in the subcutaneous injection model was the result of disturbed attachment. To better investigate the functional consequences of these changes, the invasive capacity of CMS4 cells proficient and deficient for PAK2 was analysed. When plated on extracellular matrix coated transwells, CMS4 lines effectively invade through the Matrigel whereas CMS2 cell lines failed to invade through the matrix (data not shown). Intriguingly, PAK2 KO CMS4 cells completely lost this invasive capacity (Fig. 5A, B), indicating that an activated PAK2 pathway endows MDST8 and HuTu-80 cells with migratory and invasive capacity. Similarly, primary CMS2 and CMS4 lines were generated in which PAK2 could be knocked down using a doxycycline-inducible shRNA system. PAK2 expression was clearly decreased upon knockdown compared to a non-silencing control (Supplementary Fig. S4A). As these cells grow in suspension, the impact of PAK2 knockdown on growth was not evident (Supplementary Fig. S4B). However, a clear decrease in migration through a transwell upon PAK2 knockdown was detected for the CMS4 line CONC. As reported before CMS2 lines did not display evident migration, suggesting that also primary CMS4 CRC lines were endowed with migratory capacity, which depended on the activity of PAK2 (Supplementary Fig. S4C, D). Based on these observations it is tempting to speculate that mesenchymal CRC cells, may depend on this pathway for metastasis. Previously, we reported that CRC that belong to CMS4 have a high propensity to metastasize to the peritoneal cavity, liver and lung [4, 11, 13]. To assess the impact of PAK2 deficiency on metastatic spreading of CMS4 directly, an intraperitoneal metastasis model for CRC was employed (Fig. 5C). This method previously showed high numbers of metastases with CMS4, but not CMS2 lines [11, 13]. Interestingly, PAK2 KO CMS4 lines failed to grow in the peritoneal cavity and thus failed to form metastases, while the WT counterparts efficiently induced multiple metastases, in line with the observation that PAK2 is required for outgrowth and invasion in an in vitro 3D setting (Fig. 5D). Similarly, mesenteric vein injection (Fig. 5E) of WT Dendra-labelled HuTu-80 cells displayed efficient colonization and growth of multiple metastatic foci in the liver and, due to its aggressive nature, also induced secondary seeding from the liver to the lungs with a large number of small metastases present after 4 weeks. In contrast, tdTomato-labelled PAK2 KO HuTu-80 cells were significantly less effective in forming liver metastases and almost completely failed to reseed to the lung. The limited amount of PAK2 KO cells that were detected in the lungs remained as single cells in contrast to the WT cells that grew out to form macrometastases (Fig. 5F-I; Supplementary Fig. S4E). Additionally, when injected together with WT cells, the PAK2 KO cells had a clear defect in primary and secondary seeding to metastatic sites, further proving that PAK2 was essential for the metastatic spreading of mesenchymal CRC cells to distant sites (Supplementary Fig. S4F).

## Discussion

We have shown that CRC subtypes are wired differently and contain unique subtype-specific vulnerabilities with the application of a CRISPR-Cas9 drop-out screen. Validation of the most significant drop-out in the mesenchymal subtype CMS4, PAK2, unveiled a dependency of this subtype on cytoskeletal remodeling. Moreover, loss of PAK2 hampered outgrowth, invasion and metastasis, key biological features associated with this subtype of CRC. The subtype selectivity of numerous kinases identified in the drop-out screen confirms earlier claims that CMS features can be traced to the epithelial tumor compartment [8, 10]. When scrutinizing the list of kinases identified in the screen, some expected kinases stand out. For instance, epithelial subtypes CMS2 and CMS3 are more dependent on EGFR than CMS1 and CMS4, which was shown before and further supports earlier clinical observations which suggest that CMS4 cancers do not respond to anti-EGFR therapy [41]. Similarly, AKT3 is defined as a CMS4-selective kinase, which confirms our previous findings using a bioinformatical pipeline [24]. However, most subtype-specific kinases will need more detailed mechanistic analysis to understand their selectivity, which is likely to reveal more subtype specific biological features. For PAK2, the selectivity is almost absolute as it is essential in the large majority of CMS4 lines, while it is dispensable in all tested CMS1/2/3 cell lines.

The role of PAK2 in CMS4 goes beyond survival or proliferation, which is typically revealed in a drop-out screen, as it is also crucial for regulation of signaling downstream of βPIX and attachment, and its loss leads to altered actin cytoskeleton dynamics and ultimately to a lack of invasion and metastasis. Actin remodeling and dynamics necessary for cell migration and invasion are typically regulated by Rho family GTPases. Briefly, activated RHO enhances stress fiber formation, RAC induces lamellipodia formation and CDC42 stimulates filopodia [42, 43]. This suggests that deletion of PAK2 in CMS4 cells results in a shift towards RHO activity, which is in line with earlier observations that indicate that PAK activity acts downstream of RAC/CDC42 to phosphorylate and inactivate GEF-H1, a guanine nucleotide exchange factor (GEF) for RhoA [28]. Deletion of PAK2 could break this GEF inhibitory effect, activating RhoA and allowing for RhoA-induced stress fiber formation, which could provide a model for the observed phenotypical differences in actin cytoskeleton dynamics.

Clearly, the drop-out screen results indicated that PAK1 and PAK2 are not interchangeable. Earlier work also revealed clear differences between these seemingly highly similar kinases [44]. For instance, PAK1 cannot substitute for the pro-apoptotic role exerted by PAK2 [45]. In addition, PAK2 was shown to localize to focal adhesions, while PAK1 displayed a more cytosolic staining. Under these conditions, PAK2 was suggested to mediate attachment, while PAK1 was involved in spreading [45]. In CMS4 lines it is evident that deletion of PAK2 increases cofilin phosphorylation, which lowers its actin-severing activity and thus coincides with the observed decrease in actin turnover and the relatively more stable stress fibers. Importantly, inhibition of growth appeared to depend on the necessity of cells to grow in attachment and was less evident for the primary lines that grow in suspension. Nevertheless, the impact on migration and invasion, which is a crucial first step in metastasis, was apparent in both PAK2 deficient CMS4 cell lines and primary cells and was absent from their CMS2 counterparts. This process has been reported to require active actin cytoskeleton remodeling and is directly related to RAC/CDC42 activity and downstream signaling [46]. Although the exact orchestration of this signaling pathway in CMS4 requires additional mechanistic insight, the consequences of PAK2 deletion are highly significant as a near complete inhibition of in vivo growth and metastasis was observed in multiple models. Moreover, inhibition of PAK group 1 family kinases using FRAX597 confirmed this higher dependency of CMS4 lines and organoids on PAK signaling. Combined, our data therefore highlight the potential of PAK2 as a novel target for therapy; either by preventing invasion to and colonization of metastatic sites, by preventing outgrowth of single cell metastases, or by a combined effect. Unfortunately, the only inhibitors available target all members of the PAK family I, while the current data point out that targeting PAK2 suffices. Focusing treatment solely on PAK2 might diminish unwanted side effects, while effectively inhibiting the metastatic potential of the aggressive subtype CMS4.

## Conclusions

In conclusion, we have identified PAK2 as a unique and selective essential kinase for outgrowth and metastasis of the mesenchymal subtype of CRC (CMS4) using in vitro and in vivo models. We suggest that PAK2 plays a key role in the actin cytoskeleton remodeling of CMS4 cell lines. Furthermore, we demonstrate that CMS4 cell lines and human-derived organoids are sensitive to the inhibition of PAK group 1 family kinases, suggesting a potential novel therapy for the poor-prognosis subtype of CRC.

## Supplementary Information

**Additional file 1: Supplementary Fig. S1**. Validation of PAK2 as an essential kinase for CMS4 cell lines. **A,** *PAK1–3* mRNA expression levels in a panel of 28 CRC cell lines, also including those used for the drop-out screen, as determined by quantitative PCR. Of note: diamond for *PAK3* located on x-axis indicates no mRNA could be detected in this sample. **B, C,** 2Log mRNA expression levels of *PAK4–6* in CRC cell lines (**B**) and tumors (**C**), determined by microarray or RNA sequencing. **D**, Western blot for PAK1 protein expression in HT55 & SW948 (CMS2) and HuTu-80 & MDST8 (CMS4). 2,2,2-Trichloroethanol (2,2,2TCE) signal (excerpt taken around 60 kDa region) indicates amount of protein loaded per cell line. Numbers on the left represent molecular weight in kDa according to a stained protein ladder loaded in the gel. Representative blot of *N* = 3 is displayed. **E, F,** *PAK2* (**E**) and *PAK1* (**F**) gene expression levels in cells expressing non-silencing (NS) or PAK2 targeting shRNA (PAK2-KD), as determined by qPCR. Expression was normalized to NS condition, *N* = 3, mean + S.D. is plotted. **G,** *PAK2* gene expression levels in cells expressing non-silencing (NS) or PAK2 targeting shRNA (PAK2-KD), as determined by qPCR in CMS4 cell lines not included in the initial screen (CaR-1, LS123) and a CMS4 line in which PAK2 was not a significant drop-out in the initial screen (Colo320-HSR). **H,** Relative contribution of PAK2 KD cells to co-culture in competition assay set-up, over time, in additional CMS4 cell lines. Contribution of shRNA-turboRFP expressing cells was first normalized to contribution on day 0 post sort, and subsequently calculated relative to contribution of NS shRNA-turboRFP expressing cells at each individual time-point. *N* ≥ 2, mean + s.e.m. is plotted. **Supplementary Fig. S2.** PAK2 loss alters cell morphology, not induction of apoptosis, of CMS4 cell lines**. A,** Validation of successful PAK2 knock-out (KO) in OUMS-23 CRISPR-Cas9-edited single cell knock-out clones. WT = wildtype. Numbers on the left represent molecular weight in kDa according to a stained protein ladder loaded in the gel. **B,** Phase contrast images of OUMS-23 WT and KO cells 7 days post-plating. Region of interest (ROI) selected for lower panels is indicated in white dashed box. **C, D,** Outgrowth of WT and PAK2-KO cells plated on plastic (**C**) or Matrigel (**D**) relative to day 0, as measured 4 days post-plating by CellTiter Glo. Representative experiment of *N* = 3 is shown. Data plotted is mean + S.D. N.S. = not significant, ** = *P* < 0.005, *** = *P* < 0.001, **** = *P* < 0.0001 in two-tailed, Welch-corrected Student’s T-test. **E, F, G,** Relative outgrowth of cells after 3 days of treatment with different concentrations of Oxaliplatin (**E**), 5-FU (**F**) and Paclitaxel (**G**) measured using CellTiter Glo and normalized to DMSO treated control relative to a day 0 baseline measurement. Data plotted is mean ± S.D. Representative experiment of *N* = 3 is shown. **H**, Percentage of apoptotic cells present 48 hours post plating as assessed by Nicoletti assay. Bar graphs depict mean + s.e.m., *N* = 3, n.s. = not significant as calculated by two-tailed, Welch-corrected Student’s T-test. **I,** Phase contrast images of CMS2 (HT55) and CMS4 (HuTu-80, MDST8) WT and KO cells 5 days post-plating. Scale bar represents 50 μm. **Supplementary Fig. S3**. PAK2 deficiency affects actin cytoskeleton turnover and focal adhesion localization**. A,** *ARHGEF7* (*βPIX)* gene expression levels in cells expressing non-silencing (NS) or *ARHGEF7* (*βPIX)* targeting shRNAs (Sh-1, Sh-2), as determined by qPCR. Expression was normalized to NS condition, *N* = 3, mean + s.d. is plotted. **B, C,** Region of interest stills (Fig. 4D) from time-lapse TIRF microscopy at indicated time points (min = minutes) of cell lines transduced with Lifeact-GFP (green) and paxillin-mCherry (magenta). Scale bars indicate 5 μm. See corresponding **Supplementary Movies** **5****and** **6**, available online, for the full time-lapse recording. **D, E,** Heatmaps show actin cytoskeleton dynamics over 60 minutes for HuTu-80 (**D**) and MDST8 (**E**) in a unique color per time frame. Note the rapid emergence and disappearance of filopodia in the WT condition and relative stability of filopodia over time in PAK2-KO cells. **Supplementary Fig. S4**. Metastatic colonization and secondary spread is strongly reduced by PAK2 loss**. A,** *PAK2* gene expression levels in primary cultures expressing non-silencing (NS) or PAK2 targeting shRNA (PAK2-KD), as determined by qPCR. **B**, Relative outgrowth of NS or PAK2-KD primary cell lines plated on low-adherent culture plates relative to day 0, as measured 4 days post-plating by CellTiter Glo.. Data plotted is mean ± S.D. Representative experiment of *N* = 3 is shown. **C**, **D**, Quantification (**C**) and representative images (**D**) of Migration Transwell experiments. Bar plots display relative area percentage covered by cells (purple) in Transwell image. Data plotted is mean ± S.D. Values representing *N* = 2 are plotted. **E,** Representative images of liver and lung sections stained for human Ku80 (DAB, indicated in brown), derived from two mice per condition. Along with the other Ku80-stained sections, these images were the source for quantification as included in Fig. 5G, I. **F,** Macroscopic images of tumor burden in full livers and lungs of mice injected with a 50–50% mixture of HuTu-80 WT-Dendra (green) and PAK2-KO-tdTomato cells (magenta). Pictures were taken shortly after mice were sacrificed.

**Additional file 2: Supplementary Movie 1.** Full time lapse recording of Hutu-80 WT vs. PAK2-KO cells. Cell lines were transduced with Lifeact-GFP (green) and paxillin-mCherry (magenta). Images were acquired every 30 seconds for 1–3 hours by time-lapse TIRF microscopy (NIKON Eclipse Ti) with a 60x/1.49 NA oil objective. Time stamp indicates minutes passed since the start of the recording. Selected still images and further analysis from the full time-lapse recording are represented in Fig. 4D, E and Supplementary Fig. S4B-E.

**Additional file 3: Supplementary Movie 2.** Selected ROI from full time lapse recording of Hutu-80 WT vs. PAK2-KO cells. Cell lines were transduced with Lifeact-GFP (green) and paxillin-mCherry (magenta). Images were acquired every 30 seconds for 1–3 hours by time-lapse TIRF microscopy (NIKON Eclipse Ti) with a 60x/1.49 NA oil objective. Time stamp indicates minutes passed since the start of the recording. The ROI was selected from the full time-lapse recording shown in Supplementary Movie 1.

**Additional file 4: Supplementary Movie 3***.* Full time lapse recording of MDST8 WT vs. PAK2-KO cells. Cell lines were transduced with Lifeact-GFP (green) and paxillin-mCherry (magenta). Images were acquired every 30 seconds for 1–3 hours by time-lapse TIRF microscopy (NIKON Eclipse Ti) with a 60x/1.49 NA oil objective. Time stamp indicates minutes passed since the start of the recording. Selected still images and further analysis from the full time-lapse recording are represented in Fig. 4D, E and Supplementary Fig. S4B-E.

**Additional file 5: Supplementary Movie 4**. Selected ROI from full time lapse recording of MDST8 WT vs. PAK2-KO cells. Cell lines were transduced with Lifeact-GFP (green) and paxillin-mCherry (magenta). Images were acquired every 30 seconds for 1–3 hours by time-lapse TIRF microscopy (NIKON Eclipse Ti) with a 60x/1.49 NA oil objective. Time stamp indicates minutes passed since the start of the recording. The ROI was selected from the full time-lapse recording shown in Supplementary Movie 3.

**Additional file 6: Supplementary Movie 5***.* Split channel recordings from full time lapse imaging of Hutu-80 WT vs. PAK2-KO cells. Split channel view of time lapse recording shown in Supplementary Movie 2. Cell lines were transduced with Lifeact-GFP (green) and paxillin-mCherry (magenta). Images were acquired every 30 seconds for 1–3 hours by time-lapse TIRF microscopy (NIKON Eclipse Ti) with a 60x/1.49 NA oil objective. Time stamp indicates minutes passed since the start of the recording.

**Additional file 7: Supplementary Movie 6***.* Split channel recordings from full time lapse imaging of MDST8 WT vs. PAK2-KO cells. Split channel view of time lapse recording shown in Supplementary Movie 4. Cell lines were transduced with Lifeact-GFP (green) and paxillin-mCherry (magenta). Images were acquired every 30 seconds for 1–3 hours by time-lapse TIRF microscopy (NIKON Eclipse Ti) with a 60x/1.49 NA oil objective. Time stamp indicates minutes passed since the start of the recording.

**Additional file 8: Table S1*.*** Raw normalized sgRNA counts per sample per cell line of the CRISPR-Cas9 drop-out screen performed.

**Additional file 9: Table S2.** Results from analysis of CRISPR-Cas9 drop-out screen representing the fold change within each replicate of sgRNA counts between t1 and t0.

**Additional file 10.** Full Western blot membrane images represented in the manuscript.

## Data Availability

All data generated or analyzed during this study are included in this published article and its supplementary information files.

## Abbreviations

CRC: Colorectal cancer
CMS: Consensus molecular subtype
PAK2: p21-activated kinase 2
CIN: Chromosomal instable
MSI: Microsatellite instable
gRNA: guide RNA
WT: Wildtype
PAK2-KD: PAK2 knockdown
KO: CRISPR-Cas9 knock-out
FA: Focal adhesion
TIRF: Total internal reflection fluorescence microscopy
LIMK: LIM kinase
GEF: Guanine nucleotide exchange factor
