# Supplementary material for: Subtype-specific kinase dependency regulates growth and metastasis of poor-prognosis mesenchymal colorectal cancer
Source: J Exp Clin Cancer Res. 2023 Mar 3;42:56. doi: 10.1186/s13046-023-02600-9 (PMC9983221; doi:10.1186/s13046-023-02600-9)

Supplementary Figure 1

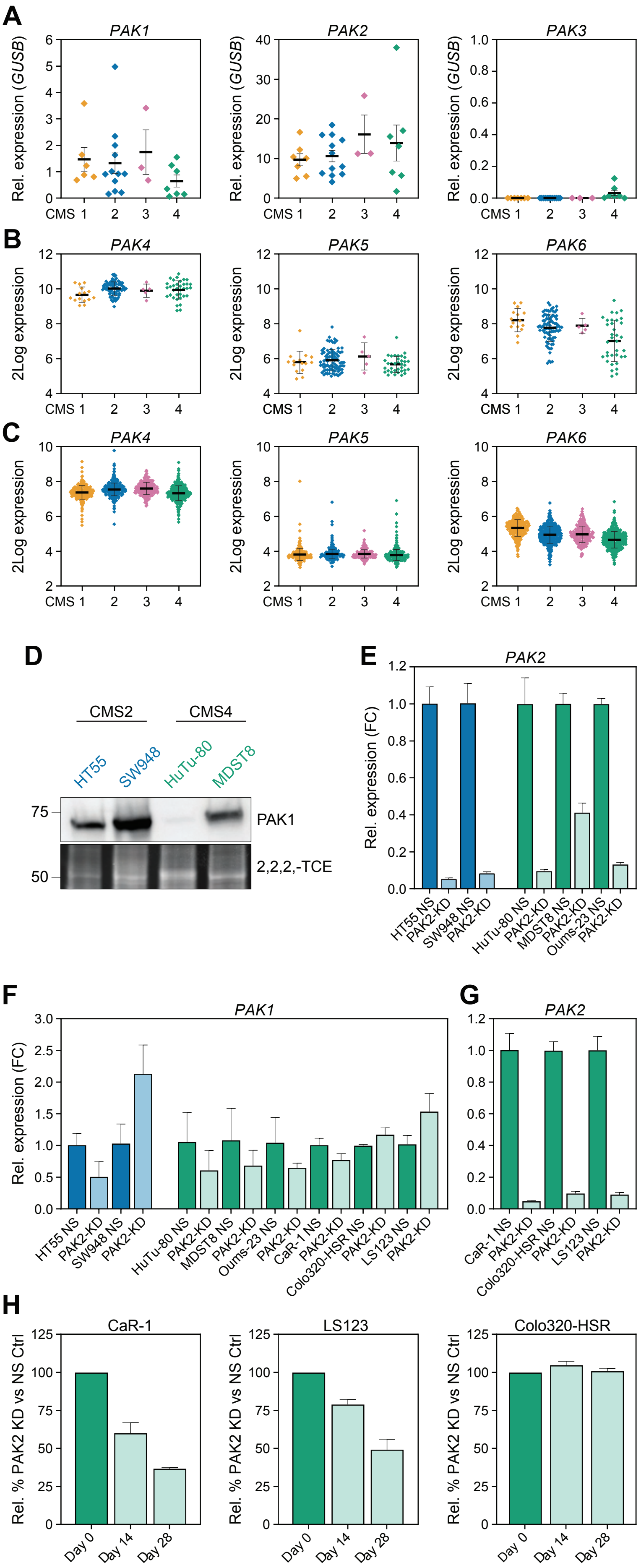

Supplementary Figure 2

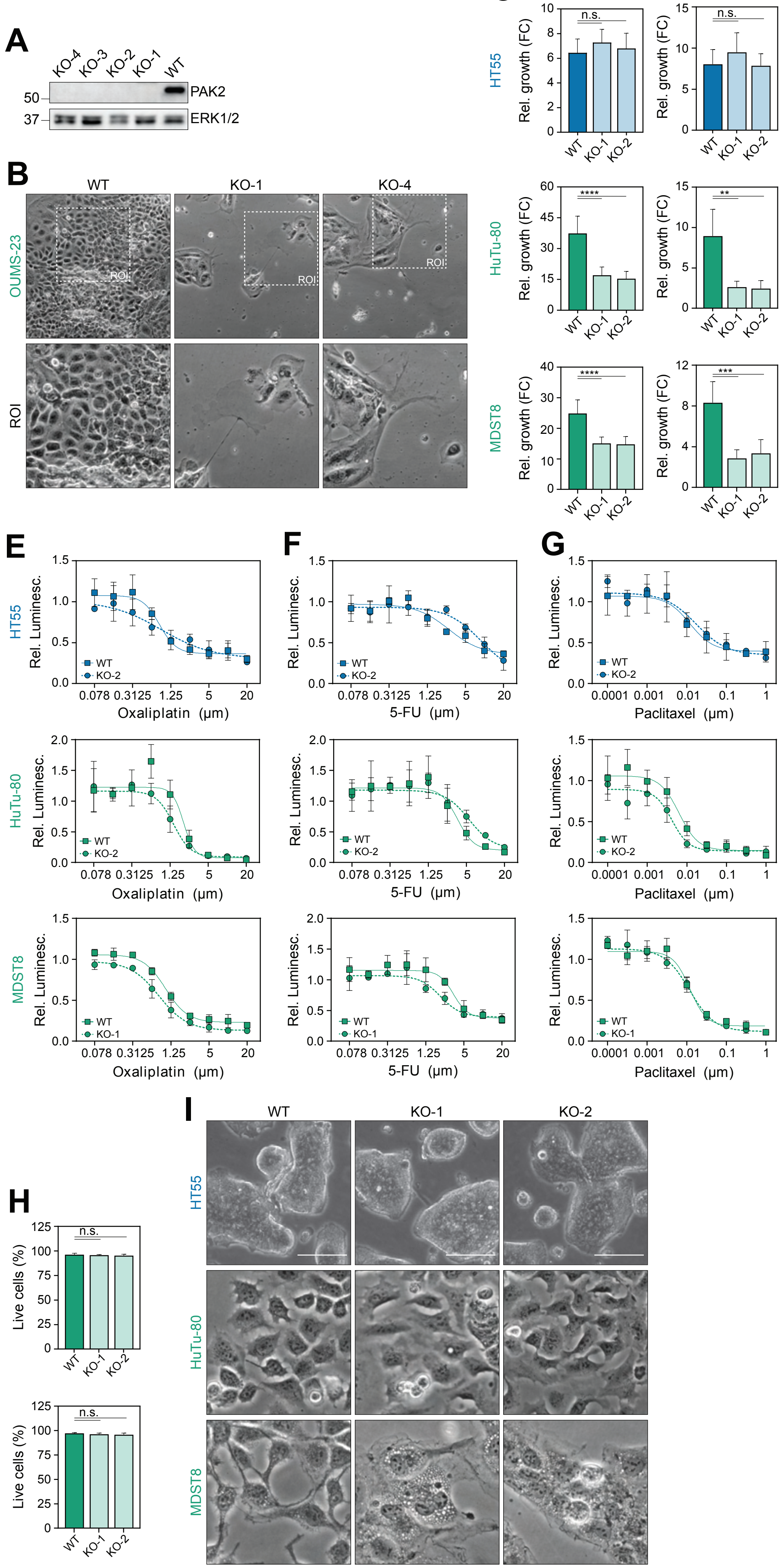

Supplementary Figure 3

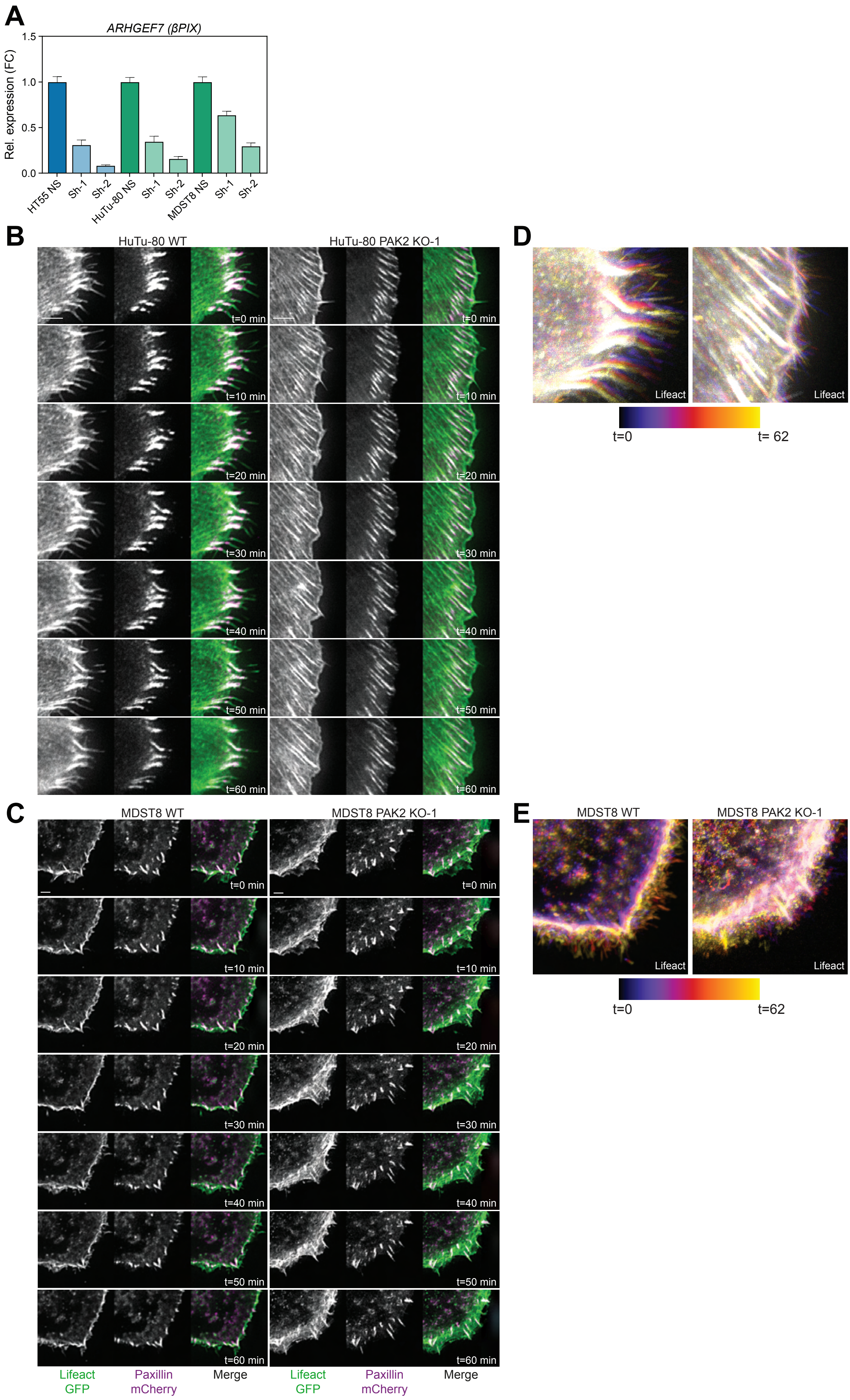

Supplementary Figure 4

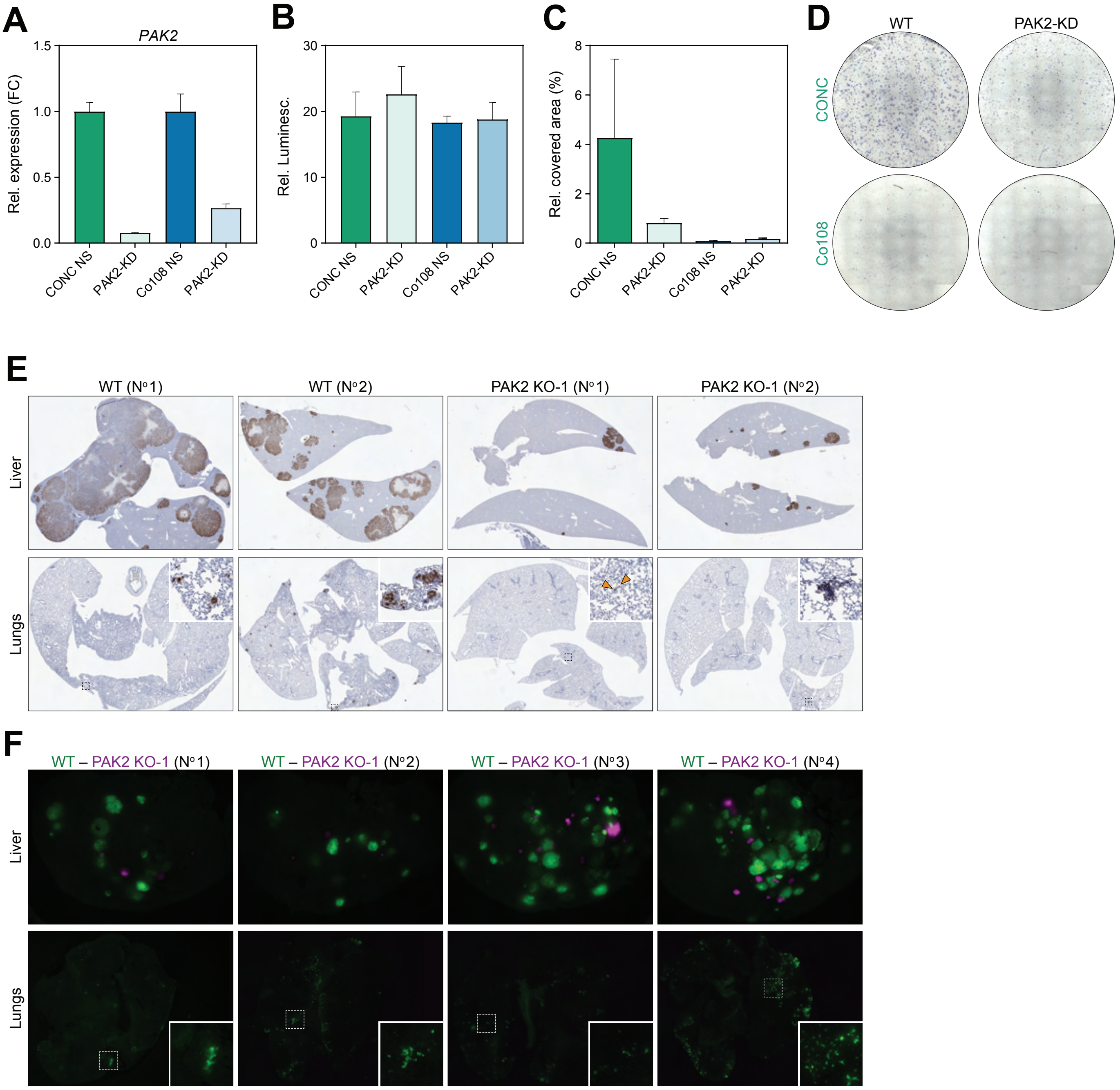

Supplement: Supplementary file 1 — Additional file 1: Supplementary Fig. S1. Validation of PAK2 as an essential kinase for CMS4 cell lines. A, PAK1–3 mRNA expression levels in a panel of 28 CRC cell lines, also including those used for the drop-out screen, as determined by quantitative PCR. Of note: diamond for PAK3 located on x-axis indicates no mRNA could be detected in this sample. B, C, 2Log mRNA expression levels of PAK4–6 in CRC cell lines (B) and tumors (C), determined by microarray or RNA sequencing. D, Western blot for PAK1 protein expression in HT55 & SW948 (CMS2) and HuTu-80 & MDST8 (CMS4). 2,2,2-Trichloroethanol (2,2,2TCE) signal (excerpt taken around 60 kDa region) indicates amount of protein loaded per cell line. Numbers on the left represent molecular weight in kDa according to a stained protein ladder loaded in the gel. Representative blot of N = 3 is displayed. E, F, PAK2 (E) and PAK1 (F) gene expression levels in cells expressing non-silencing (NS) or PAK2 targeting shRNA (PAK2-KD), as determined by qPCR. Expression was normalized to NS condition, N = 3, mean + S.D. is plotted. G, PAK2 gene expression levels in cells expressing non-silencing (NS) or PAK2 targeting shRNA (PAK2-KD), as determined by qPCR in CMS4 cell lines not included in the initial screen (CaR-1, LS123) and a CMS4 line in which PAK2 was not a significant drop-out in the initial screen (Colo320-HSR). H, Relative contribution of PAK2 KD cells to co-culture in competition assay set-up, over time, in additional CMS4 cell lines. Contribution of shRNA-turboRFP expressing cells was first normalized to contribution on day 0 post sort, and subsequently calculated relative to contribution of NS shRNA-turboRFP expressing cells at each individual time-point. N ≥ 2, mean + s.e.m. is plotted. Supplementary Fig. S2. PAK2 loss alters cell morphology, not induction of apoptosis, of CMS4 cell lines. A, Validation of successful PAK2 knock-out (KO) in OUMS-23 CRISPR-Cas9-edited single cell knock-out clones. WT = wildtype. Numbers on th [file 13046_2023_2600_MOESM1_ESM.pdf]
