# Supplementary material for: Subtype-specific kinase dependency regulates growth and metastasis of poor-prognosis mesenchymal colorectal cancer
Source: J Exp Clin Cancer Res. 2023 Mar 3;42:56. doi: 10.1186/s13046-023-02600-9 (PMC9983221; doi:10.1186/s13046-023-02600-9)
Supplement: Supplementary file 10 — Additional file 10. Full Western blot membrane images represented in the manuscript. [file 13046_2023_2600_MOESM10_ESM.pdf]

PAK1

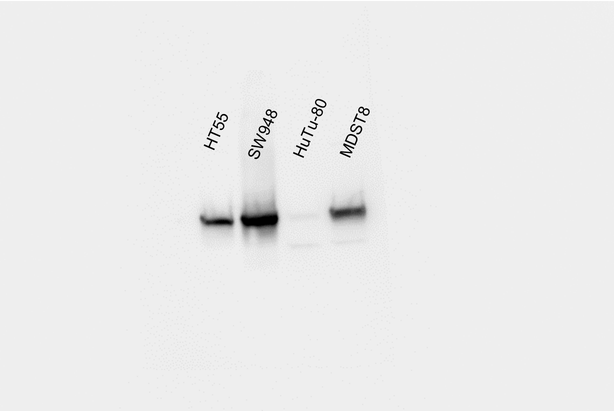


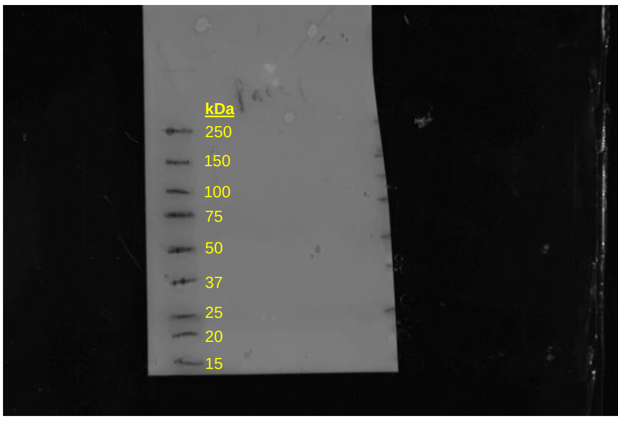


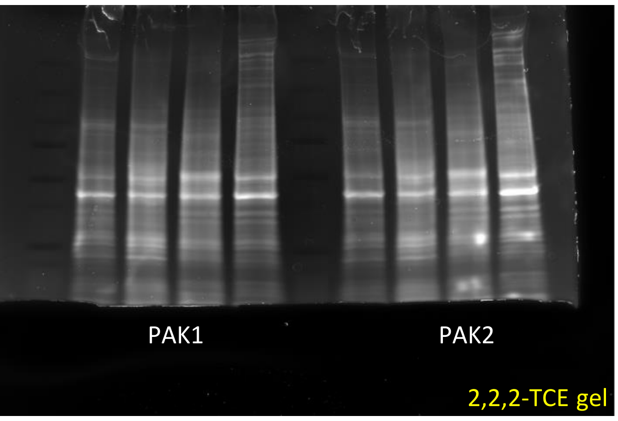


PAK2

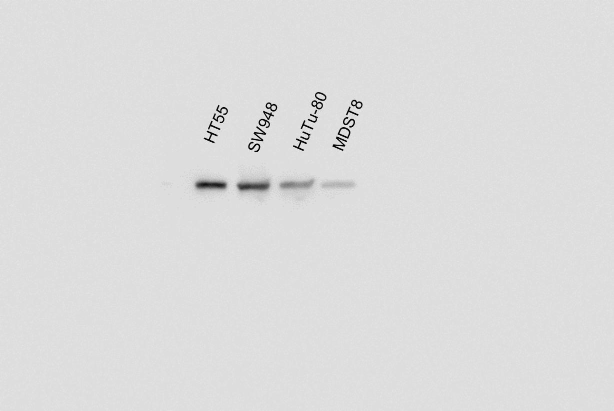


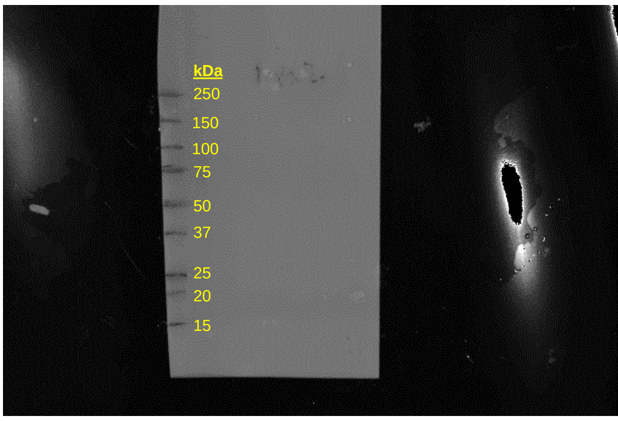


**Whole blots corresponding to Figure 2C**

**HuTu-80**

PAK1

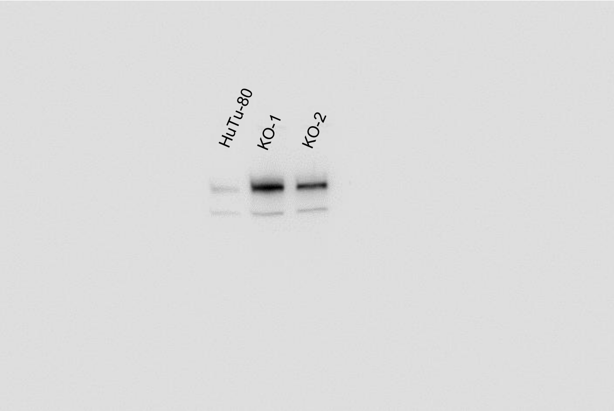


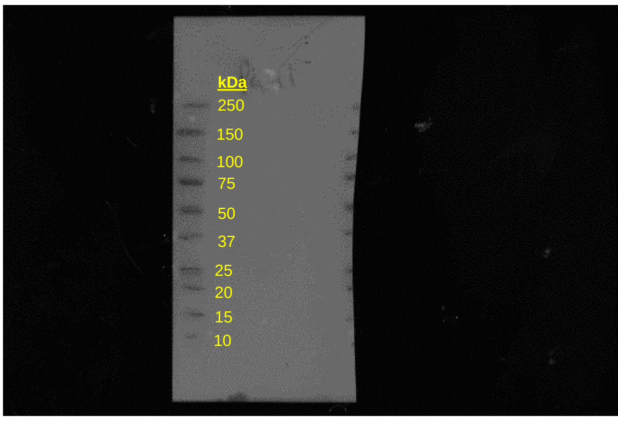


GAPDH

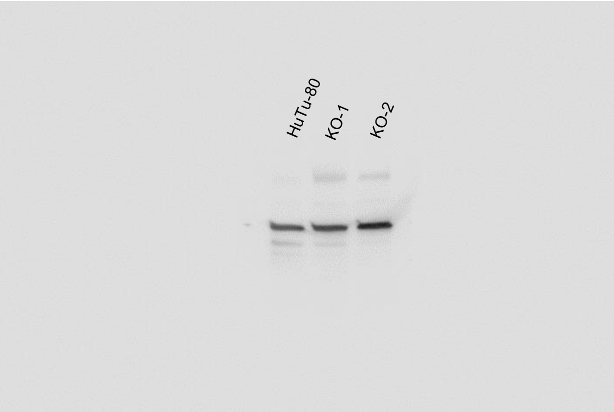


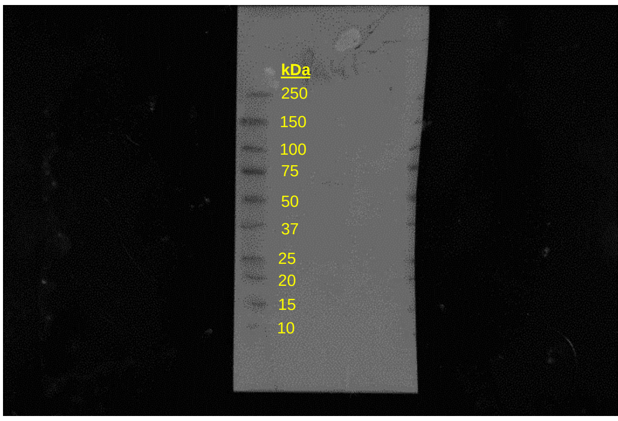


PAK2

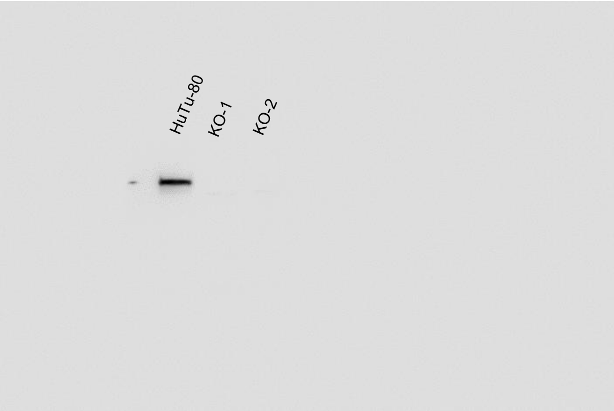


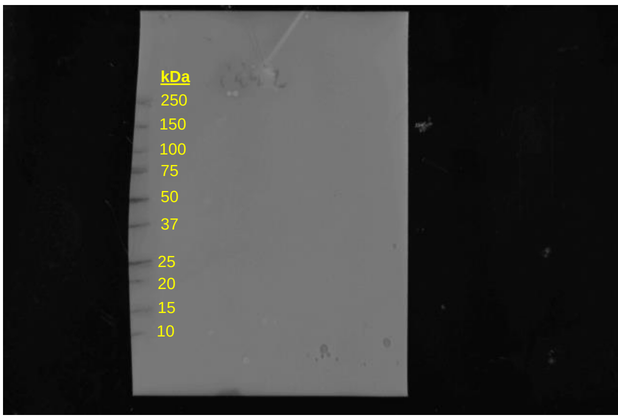


GAPDH

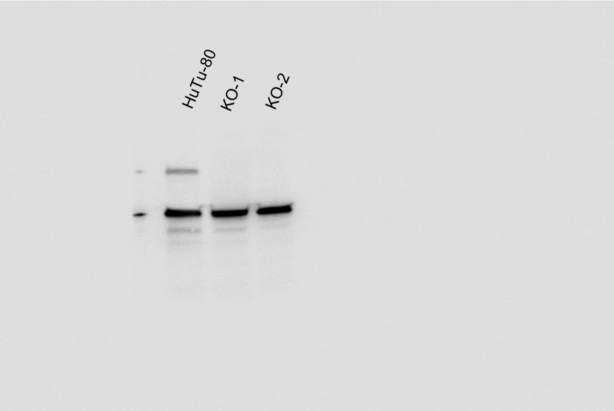


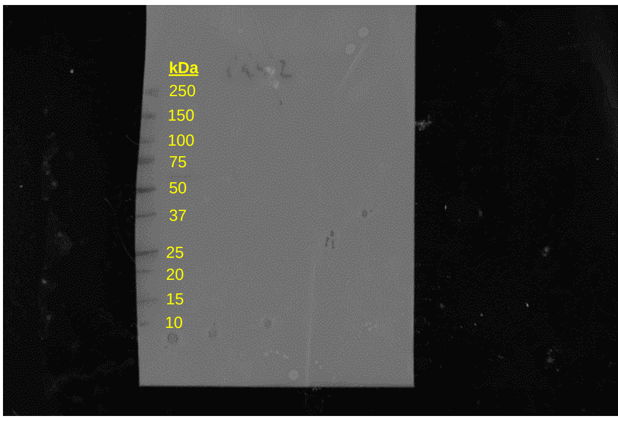


**Whole blots corresponding to Figure 3A**

**MDST8**

PAK1

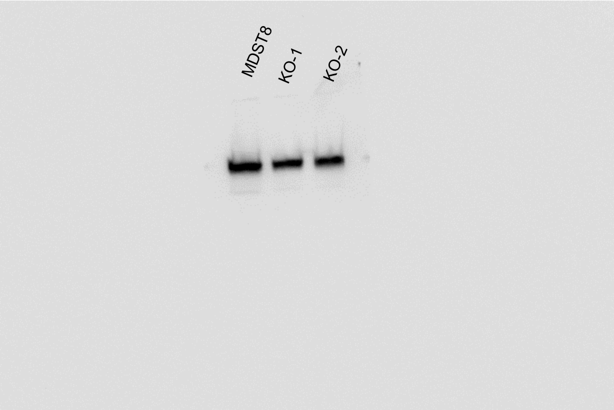


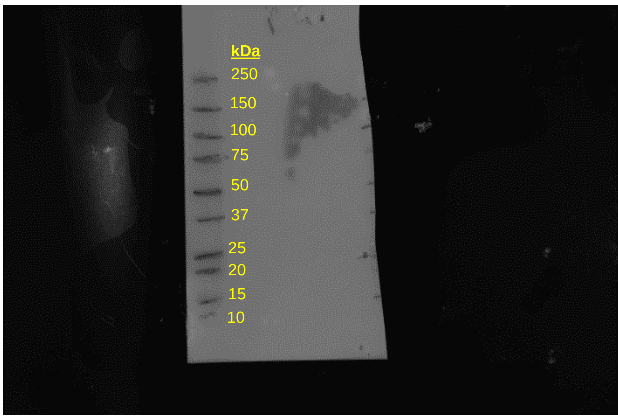


GAPDH

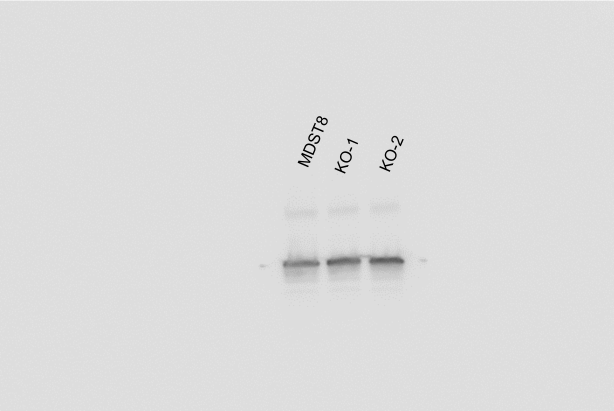


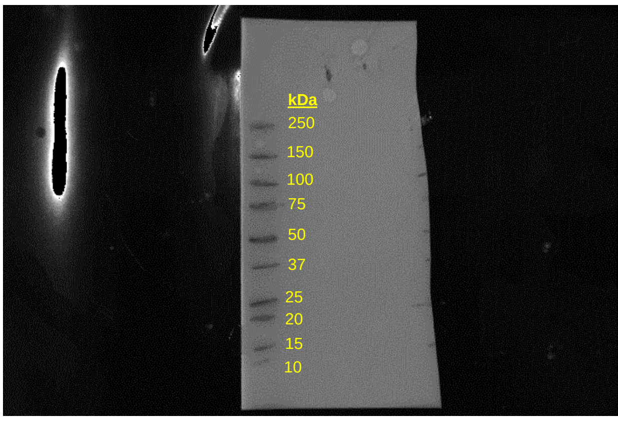


PAK2

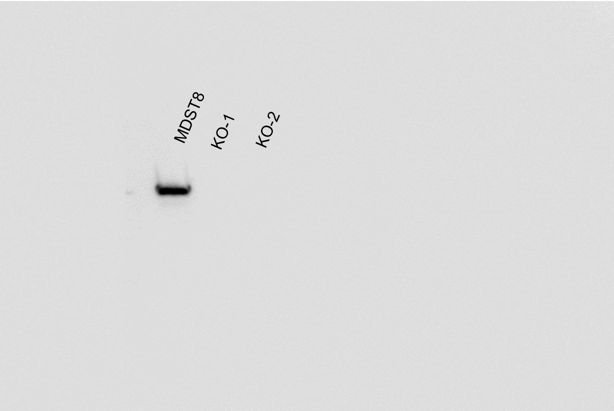


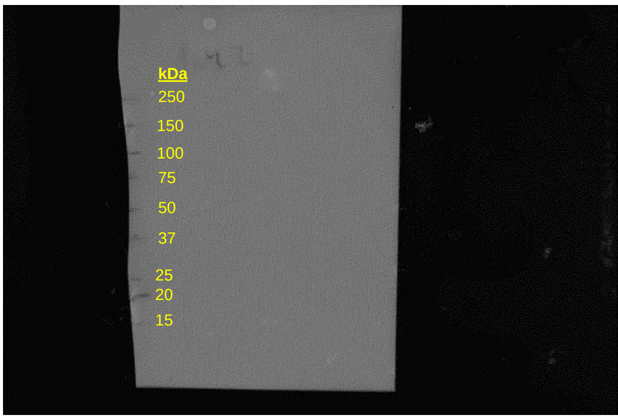


GAPDH

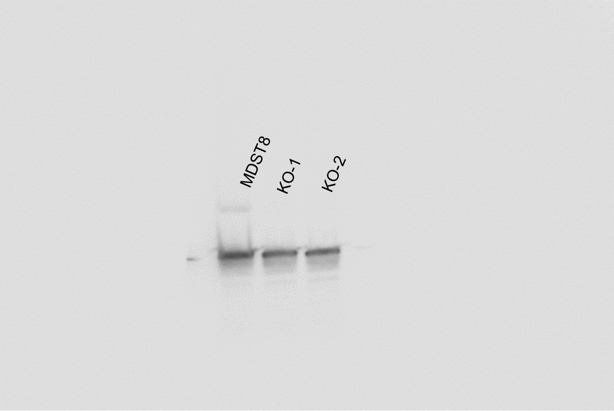


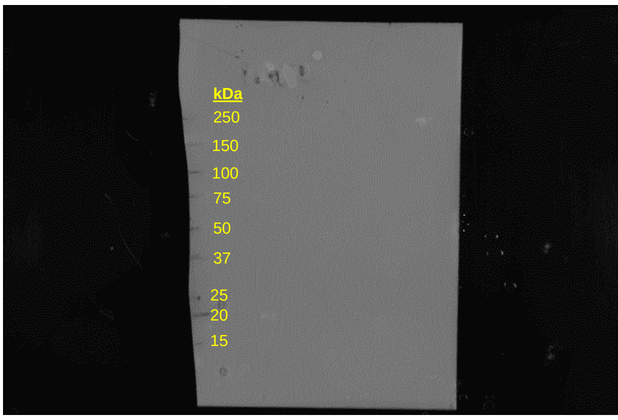


**Whole blots corresponding to Figure 3A**

**HT55**

PAK1

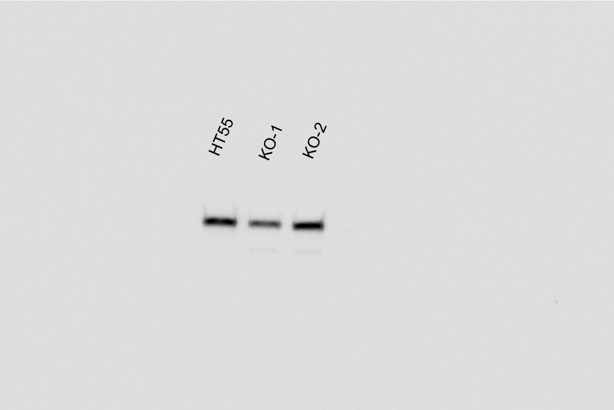


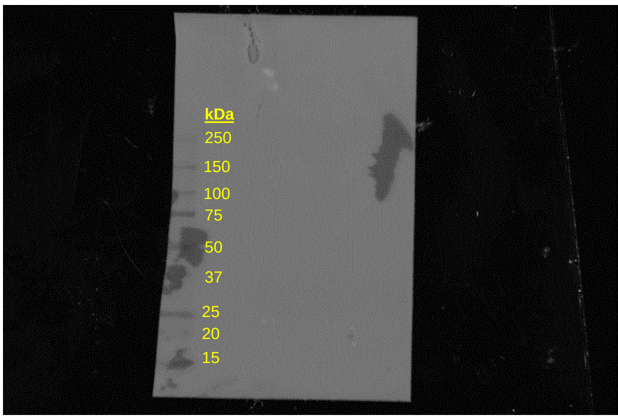


GAPDH

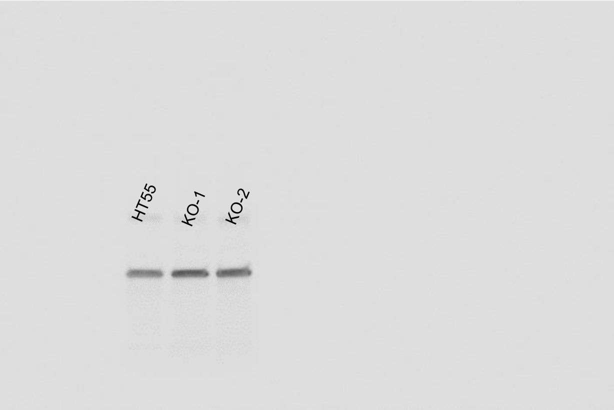


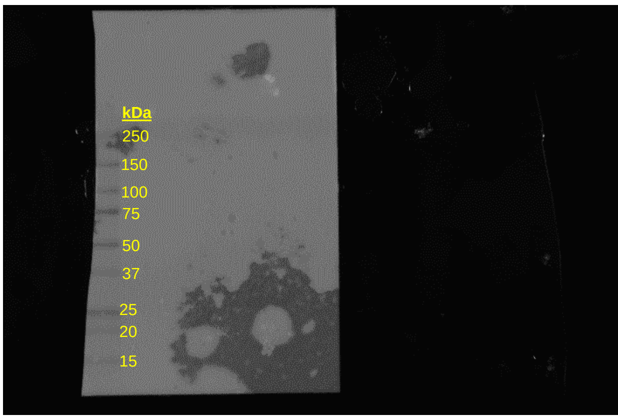


PAK2

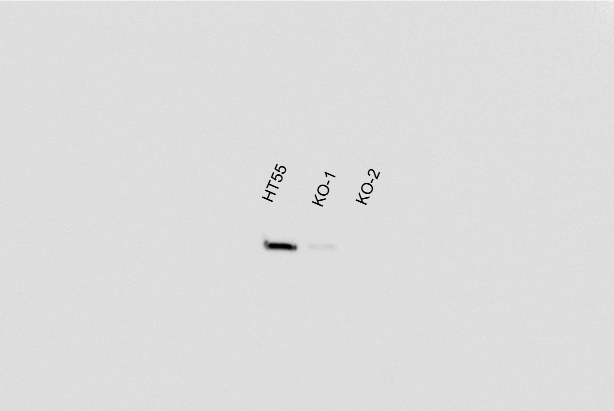


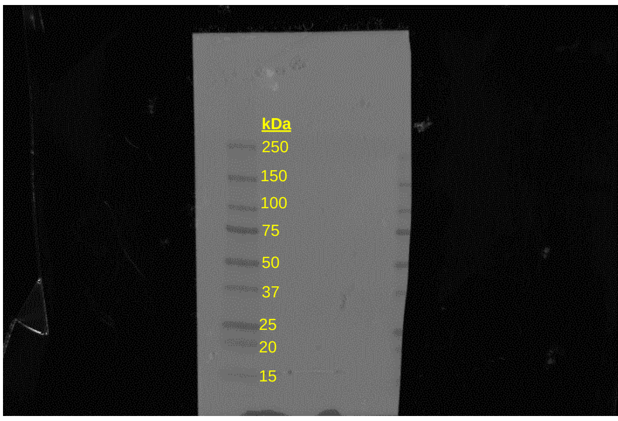


GAPDH

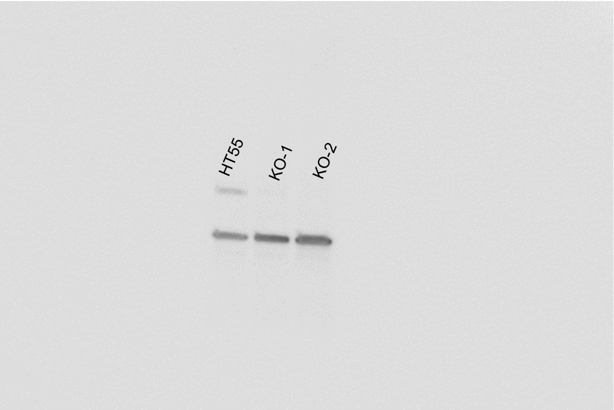


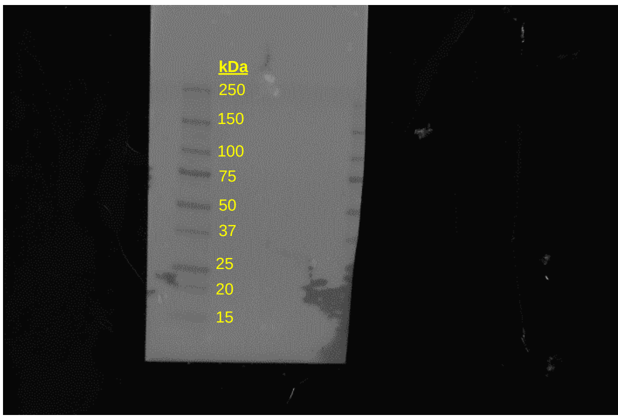


**Whole blots corresponding to Figure 3A**

P-Cofilin

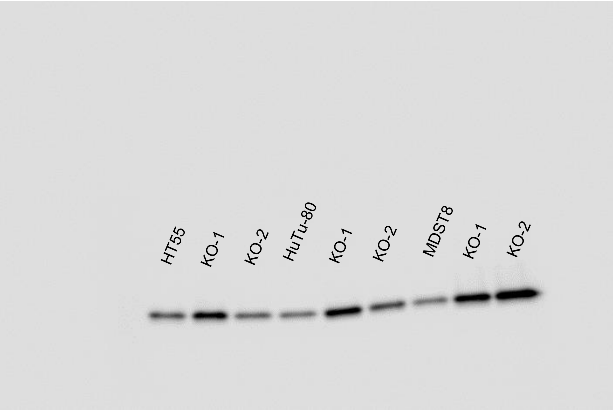


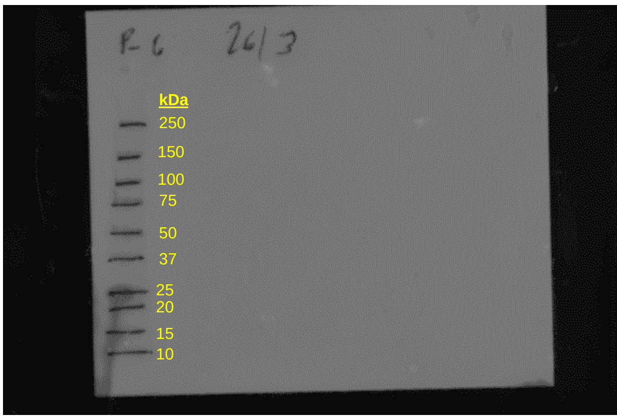


GAPDH

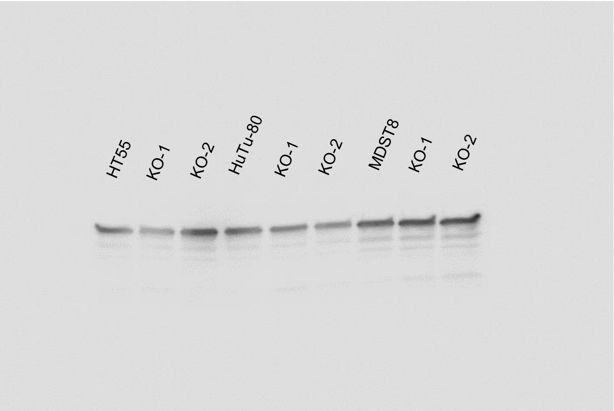


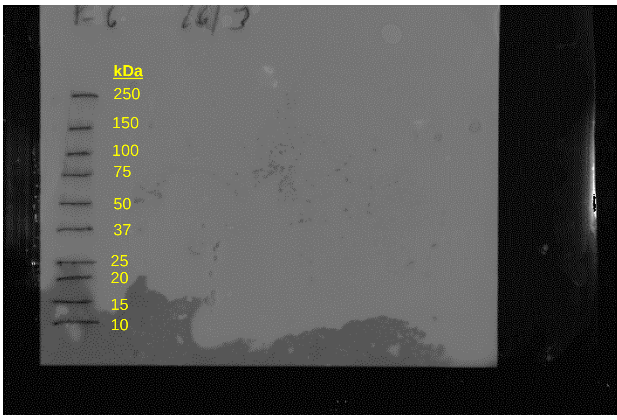


Cofilin

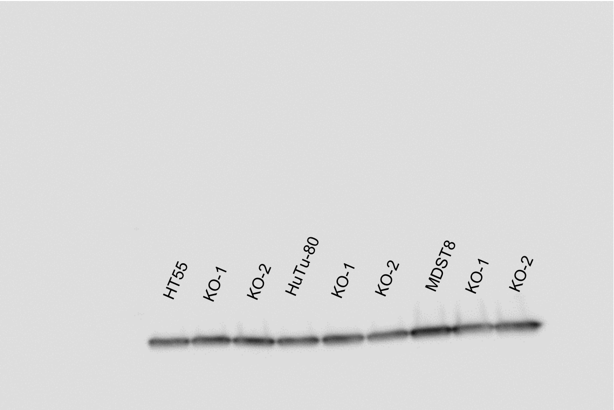


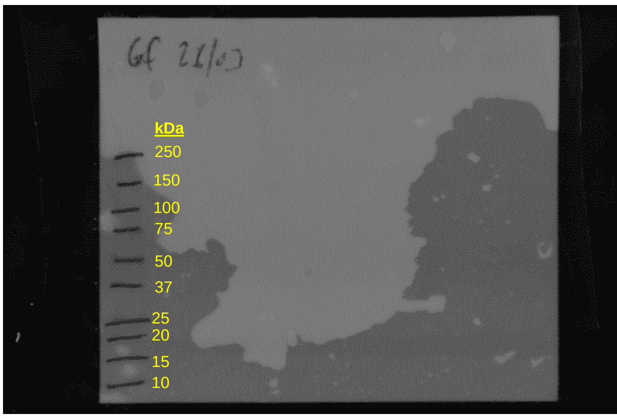


GAPDH

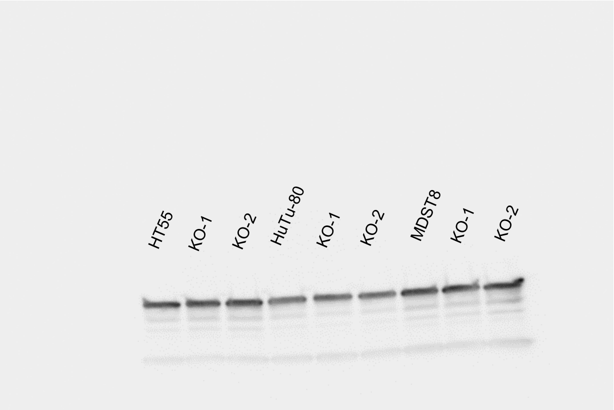


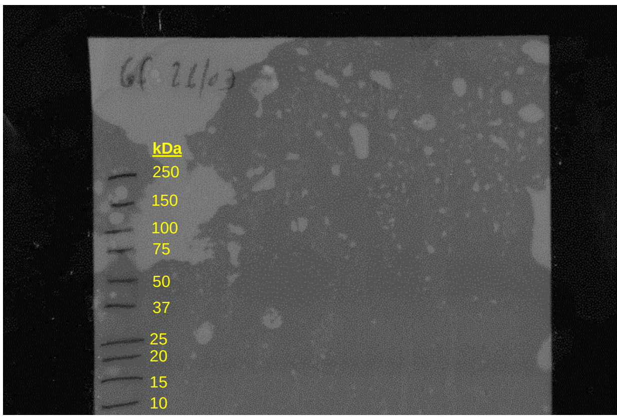


**Whole blots corresponding to Figure 4F**
